# Morphology, Crystallinity, and Electrical Performance of Solution-Processed OFETs Based on a DPP-Based Small Molecule

**DOI:** 10.1021/acsaelm.5c00731

**Published:** 2025-07-15

**Authors:** Enrique Caldera-Cruz, Reshma Raveendran, Yevhen Karpov, Felix Talnack, Brigitte Voit, Anton Kiriy, Stefan C. B. Mannsfeld

**Affiliations:** † 28408 Leibniz Institute of Polymer Research Dresden, Hohe Straße 6, Dresden 01069, Germany; ‡ Center for Advancing Electronics Dresden and Faculty of Electrical and Computer Engineering, 9169 Technische Universität Dresden, Helmholtzstraße 18, Dresden 01069, Germany; § Novaled GmbH Dresden, Elisabeth-Boer-Strasse 9, Dresden 01099, Germany; ∥ beeOLED GmbH, Niedersedlitzer Strasse 75c, Dresden 01257, Germany; ⊥ Organic Chemistry of Polymers, Technische Universität Dresden, Dresden 01062, Germany

**Keywords:** solution-processable semiconductors, diketopyrrolopyrrole, OFET, crystallinity, GIWAXS, solution-shearing, mobility

## Abstract

In this study, a diketopyrrolopyrrole (DPP)-based small molecule, 3,6-bis­(5′-(2-octyldodecyl)-[2,2’-bithiophen]-5-yl)-2,5-dipropyl-2,5-dihydropyrrolo­[3,4-*c*]­pyrrole-1,4-dione (DBT-I), was synthesized via Stille coupling. The thin-film morphology, crystallinity, and organic field-effect transistor (OFET) performance of DBT-I were systematically investigated under various solution-processing techniques and solvent environments. Particular emphasis was placed on understanding the influence of processing conditions on film microstructure and their correlation with device performance. It was found that the appropriate combination of solvent, deposition method, and thermal treatment significantly enhances the crystallinity of the DBT-I thin films. Since the electrical properties are influenced by the chemical structure and morphology of the semiconductor, the obtained low-bandgap material was integrated into OFETs fabricated by solution-processing methodologies, such as spin coating and solution-shearing. The optimal performance in OFETs was achieved with the shear-coated DBT-I films, showcasing a mobility of 0.26 cm^2^ V^–1^ s^–1^ and a current on–off ratio of 10^6^. The OFET performance correlated with morphology features such as crystallinity and grain size opens the possibility for a rational optimization of OFET characteristics.

## Graphical abstract

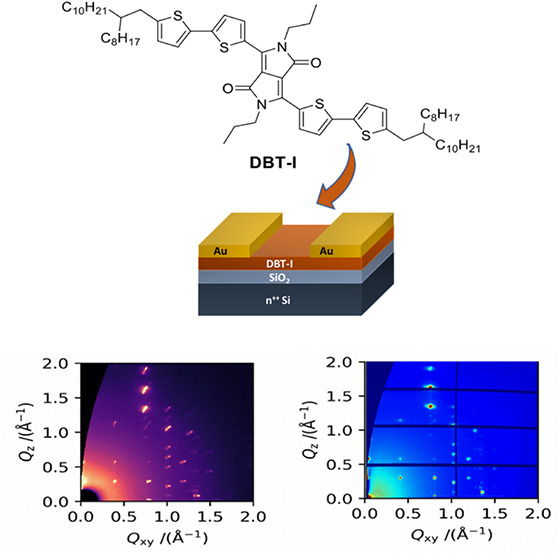

## 1 Introduction

The recent advances in solution-processed organic field-effect transistors (OFETs) have enabled the production of large-area processing of flexible electronics with applications such as e-paper displays, radio frequency identification tags, and chemical and biological sensors.^1–4^

The performance of OFETs largely depends, among other factors, on the quality of the deposited active layer and the dielectric/semiconductor interface. The chemical structure and intermolecular packing determine the reorganization energy and transfer integral that govern charge transport,^5^ while charge-carrier accumulation and transport mainly occur at the insulator/semiconductor interface. Thus, there is a strong motivation to develop solution-processable materials with enhanced molecular packing. Due to their well-defined structure, high order in the solid state, and ease of purification, small molecular materials modified featuring solubilizer side-chains are strong candidates for high-mobility OFETs. Anthony et al. reported the functionalization of pentacene and anthradithiophene with the trialkylsilylethynyl group at the central 6- and 13-positions.^6^ Solution deposition of triisopropylsilylethynyl (TIPS) pentacene yields a two-dimensional (2D) “bricklayer” arrangement in the solid state with large cofacial π-stacking in the plane of the films with the side chains oriented near normal to the substrate surface and led to OFET devices with hole mobility exceeding 1 cm^2^ V^–1^ s^–1^.^7^ Ebata et al. reported a series of 2,7-dialkylated derivatives of the conjugated core [1]­benzothieno­[3,2-*b*]­[1]­benzothiophene (BTBT) with different chain lengths. Spin-coated films showed a “layer-by-layer” structure consisting of alternately stacked aliphatic layers and BTBT core layers, where the molecules adopt a herringbone packing, facilitating 2D carrier transport properties.^8^ Further optimization of C_8_-BTBT deposition by inkjet printing led to OFETs with average carrier mobilities as high as 16.4 cm^2^ V^–1^ s^–1^.^9^ Similarly, end-group alkylated oligothiophenes (π–π functionalized) show highly ordered molecular packing in thin films and performance dependence on the chain length rather than on the number of conjugated thiophene units in the core.^10,11^ Diketopyrrolopyrrole (DPP) is a well-studied moiety of low-bandgap donor–acceptor copolymers with FET applications,^12^ as well as small molecules utilized for bulk heterojunction organic photovoltaics (OPVs),^13–16^ OFETs,^17,18^ and sensors.^19,20^ In such cases, the solubilizing alkyl chains are featured as substituents at the N-position of DPP in the backbone. Nevertheless, the role played by the alkyl side chains goes beyond simply improving the solubility of the molecules. Depending on their length, degree of branching, or heteroatoms, such aliphatic groups can modify the intermolecular packing of the material by means of π–π-stacking and crystallinity, thus affecting the mobility.^21,22^ In this regard, we present herein the synthesis of the new molecule DBT-I (3,6-bis­(5′-(2-octyldodecyl)-[2,2’-bithiophen]-5-yl)-2,5-dipropyl-2,5-dihydropyrrolo­[3,4-*c*]­pyrrole-1,4-dione), which features a DPP core functionalized with solubilizing alkyl chains at the α–ω positions. A systematic study of the correlation between the morphology and performance of the solution-processed OFETs is also discussed.

## 2 Results and Discussion

### 2.1 Design and Synthesis

In order to obtain high-performance materials for OFETs, the interchain organization is crucial. Factors such as chain orientation and backbone coplanarity play a significant role in the charge transport of the organic film.^23^ With only one α-hydrogen atom, thiophene can adopt a nearly coplanar orientation with favorable sulfur–oxygen interactions.^12^ In consequence, thiophene units were chosen to complement the DPP core, leading to the molecule DBT-I, depicted in Figure 1. This compound features solubilizing alkyl chains at the α- and ω-positions, while the N-positions of the DPP are substituted by short *n*-propyl chains, aiming to enhance molecular packing in the film-state. DBT-I is then the small molecule analogous of the well-studied polymer, poly­(diketopyrrolopyrrole-terthiophene) (PDPP3T),^24–27^ first reported in 2009 by Bijleveld et al.^28^

**Figure 1:**
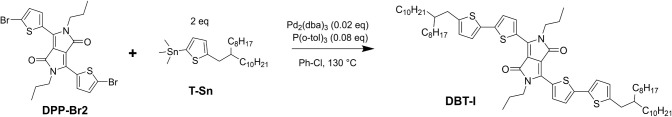
Synthesis of DBT-I by Stille coupling.

The synthesis of DBT-I started with the production of the **DPP-H** core by the succinic method, following a procedure from the literature.^29^ N-alkylation of **DPP-H** with *n*-propyl bromide yielded the precursor **DPP-nPr**, which upon subsequent bromination with NBS led to the starting material **DPP-Br**_**2**_ (Figure S1a). On the other hand, the treatment of 2-bromothiophene with *n*-butyllithium (*n*-BuLi) and 9-(iodomethyl)­nonadecane yielded the alkylated thiophene **T-C**_**20**_. Further functionalization with *n*-BuLi followed by trimethyltin chloride yielded the stannilated thiophene **T-Sn** (Figure S1b). The compound DBT-I was finally synthesized by coupling **DPP-Br**_**2**_ with two equivalents of **T-Sn** via the Stille reaction, as illustrated in Figure 1. Detailed reaction conditions can be found in the Supporting Information.

The short *n*-propyl chains of **DPP-Br**_**2**_ conferred limited solubility; thus, the reaction was carried out in chlorobenzene at 130 °C. The occurrence of the coupling could be optically corroborated by a color change in the reaction mixture from deep magenta to dark blue within the first minutes after the addition of the catalyst. After purification by flash chromatography, the product DBT-I was obtained as a dark blue, waxy film of high purity, as shown by the proton and carbon nuclear magnetic resonance spectra (^1^H NMR and ^13^C-NMR, respectively) (Figure S1c,d).

Due to the long aliphatic chains at the α- and ω-positions, DBT-I showed a high solubility in common organic solvents, such as chloroform (CHCl_3_), dichloromethane, chlorobenzene, tetrahydrofuran (THF), and toluene at room temperature.

### 2.2 Physical and Electrochemical Characterization

The optical properties of DBT-I were analyzed by ultraviolet–visible spectroscopy (UV–vis spectroscopy) in a chloroform solution and thin film, as shown in Figure 2a.

**Figure 2:**
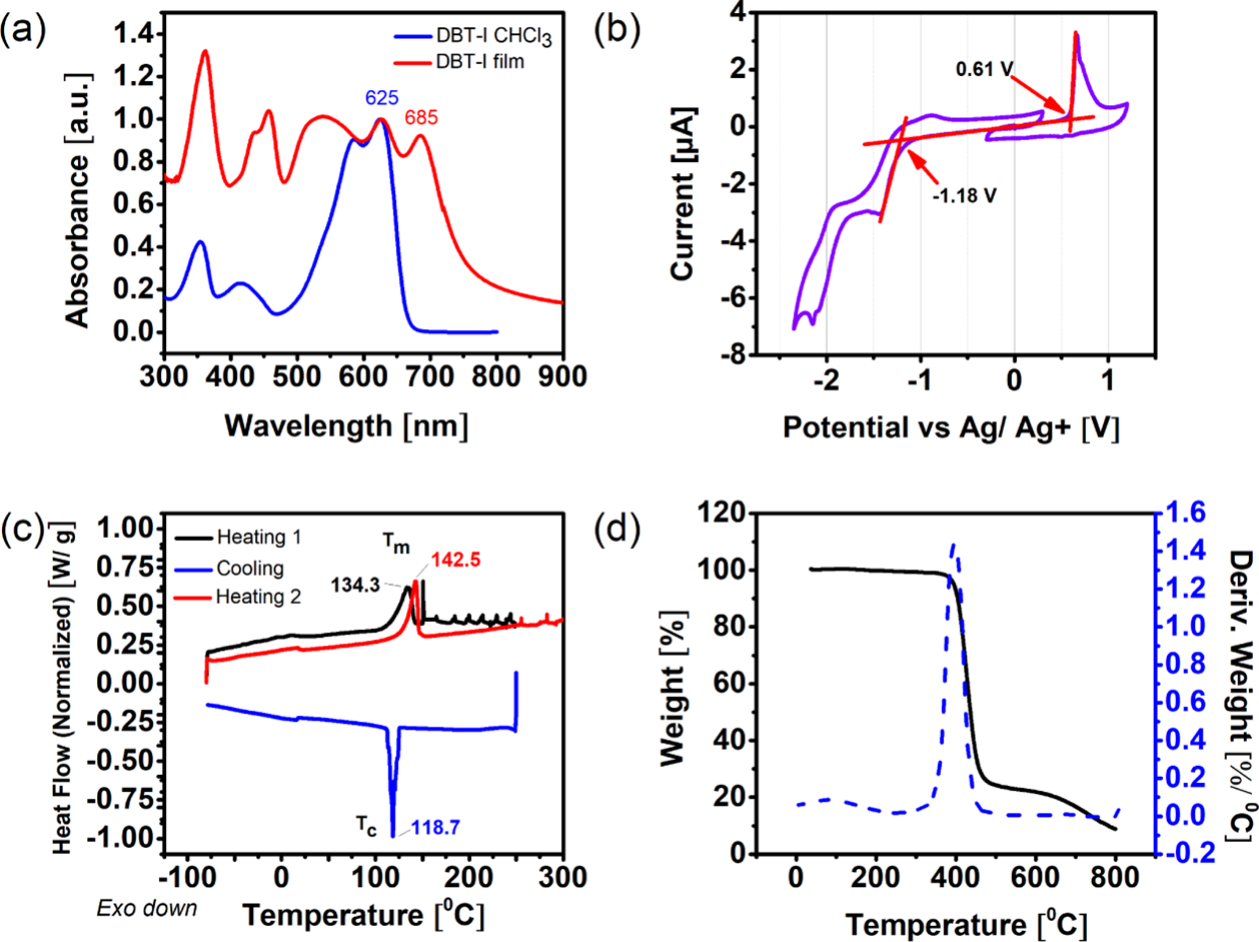
(a) Comparison of UV–vis absorption of DBT-I in chloroform solution (blue) and in a thin film (red). (b) Cyclic voltammogram in 0.1 M acetonitrile/n-N­(Bu)_4_PF_6_ solution at a 50 mV/s scan rate. (c) DSC and (d) TGA plots of DBT-I.

The chloroform solution presented the main absorption in the visible region, between 500 and 650 nm, with a wavelength of maximum absorbance (λ_max_) at 625 nm. The absorption onset was located at 663 nm, which corresponds to an optical gap $E_{g,sol}^{opt}$ of 1.87 eV. Notably, the spectrum in the thin film produced by drop-casting on glass substrates deferred significantly from its solution counterpart. The film presented the appearance of new bands that extended into the near-infrared (IR) region, with a peak at 682 nm. This suggests that the enhanced intermolecular interactions present in the solid state originate new transitions that are absent in the isolated molecules found in solution. Considering these new absorption bands, the optical gap in the film $E_{g,film}^{opt}$ corresponds to 1.63 eV (see Table 1).

**Table 1:** Optoelectronic Properties of DBT-I

	HOMO (eV)	LUMO (eV)	$E_{g,sol}^{opt}$	$E_{g,film}^{opt}$	$E_{g}^{EC}$
DBT-I	–5.59	–3.80	1.87	1.63	1.79

The energy of the frontier orbitals was determined by cyclic voltammetry (CV), as shown in Figure 2b. A nonreversible oxidation peak was readily identified at 0.66 V. By contrast, the cathodic peak was less pronounced, suggesting that the reduction process is less favored. The oxidation and reduction onsets were localized at 0.61 and −1.18 V, respectively. Assuming the redox potential of ferrocene used as an internal standard is −5.09 eV,^30^ the energy of the highest occupied molecular orbital (HOMO) was calculated as −5.59 eV, while that of the lowest unoccupied molecular orbital (LUMO) was −3.80 eV, resulting in an electrochemical gap $\left(E_{g}^{EC}\right)$ of 1.78 eV (Table 1).

The occurrence of different gap values arises from different measurement techniques. While the measurement in solution does not resemble the molecular environment of the material in the solid state, the calculated optical gap in the film state is known to underestimate the real value due to Coulombic forces that hold the exciton electrostatically bound after photoexcitation,^31^ as observed in DPP-based polymers.^23,32^ Since the assessment of $E_{g}^{EC}$ involves no excitons, this value will be taken into account for device integration.

To investigate the thermal transitions of the material, differential scanning calorimetry (DSC) and thermogravimetric analysis (TGA) were performed. Due to its long aliphatic chains and amorphous appearance in the solid state, only a glass transition temperature (*T*_g_) was initially expected. However, the DSC clearly showed reversible thermal transitions, as shown in Figure 2c. The endothermic peak at 134.3 °C was replicated at 142.5 °C in the second heating scan that followed a cooling process, which in turn showed a sharp exothermic peak at 118.7 °C. These peaks were attributed to crystallization (*T*_c_) and melting points (*T*_m_), which indicate high crystallinity. The TGA plot of Figure 2d shows the loss of 8% of the mass at 230 °C, which corresponds to the loss of both *n*-propyl side chains and the full decomposition starting at 350 °C. Thus, DBT-I is highly stable at the range of temperatures required for the annealing necessary to investigate the crystallinity.

### 2.3 Thin-Film Morphological Characterization

In this study, we compare DBT-I films deposited using solution-shearing and spin-coating methods^33–35^ to evaluate their morphological and crystalline characteristics and utilize them as active layers in OFETs. Thin-film morphological characterization of DBT-I films deposited using spin-coating and solution-shearing methods (Figure S2) was conducted using polarized optical microscopy (POM) and atomic force microscopy (AFM) techniques. This study evaluates various factors affecting the morphological features of the film, including the choice of solvents for DBT-I (chloroform and toluene), different annealing conditions: with and without elevated substrate temperature during the coating, as-coated (unannealed) and with postcoating annealing, the influence of self-assembled monolayers (SAMs), and varying shearing speeds.

Being an anisotropic material, the POM analysis of the DBT-I film provides detailed information about optical anisotropy and molecular orientation over larger areas, useful for studying the variation of crystalline structures and textures in DBT-I at different processing conditions, whereas, AFM provides high-resolution, three-dimensional topographical images of the surface at the nanoscale and enables quantitative measurements of surface roughness and texture of the film.

### 2.4 POM of Spin-Coated DBT-I Films

On the left of Figure 3 are the POM images of the spin-coated films of DBT-I, which illustrate the influence of annealing and solvent selection on the morphology of spin-coated films of DBT-I on Si/SiO_2_ substrates. The spin-coated film, without annealing, utilizing chloroform as the solvent, exhibits a remarkably uniform appearance with no discernible morphological characteristics (Figure 3a). Conversely, upon annealing at 100 °C, the film manifests small grains of roughly 0.1 μm in size with distinct molecular orientations (Figure 3b). In contrast, the DBT-I film produced through spin-coating using a toluene solution already exhibits small granular features (size ∼0.2 μm) prior to annealing (Figure 3c). Subsequent annealing at 100 °C leads to the formation of larger grains of size ∼1–2 μm (Figure 3d). Comparable morphological characteristics, characterized by slightly differing domain sizes, are evident when depositing the films onto Si/SiO_2_ wafers that have been treated with SAMs, such as phenyltrichlorosilane (PTCS) and octadecyltrichlorosilane (OTS-18) (Figure S3).

**Figure 3:**
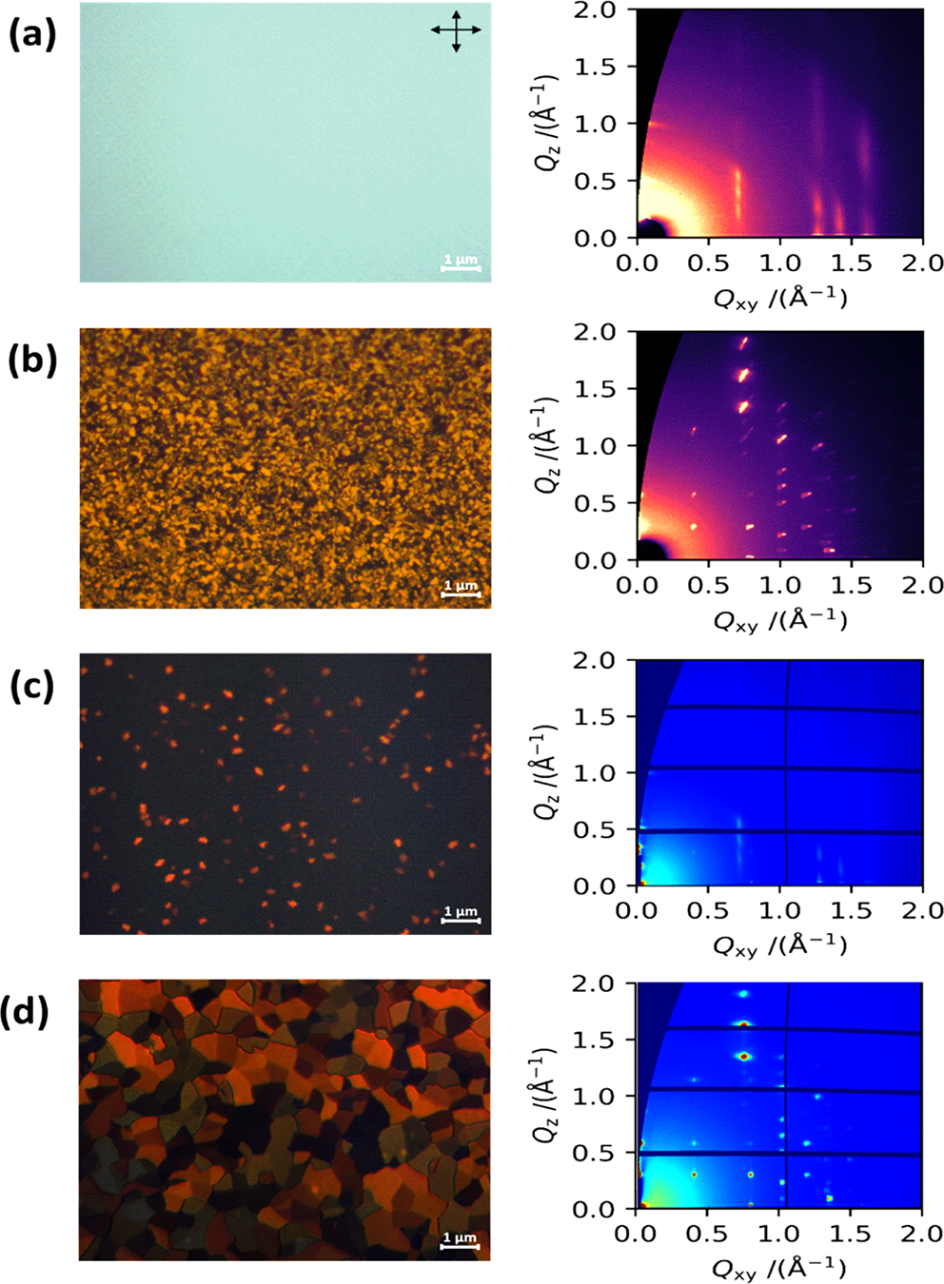
Left: polarized optical microscopy (POM) images. Right: grazing incidence wide angle X-ray scattering (GIWAXS) patterns of spin-coated films of DBT-I on SiO_2_. (a, c) Unannealed and (b, d) annealed (at 100 °C) films using the solvents (a, b) chloroform and (c, d) toluene. POM image scale: 1 μm.

### 2.5 POM of Solution-Sheared DBT-I Films

The application of the solution-shearing method yields distinctly disparate morphological characteristics on the DBT-I films in comparison to the spin-coated films, particularly when employing toluene as the solvent. Figure 4 displays the POM images of toluene-based films deposited on PTCS substrates at room temperature. The films depicted in Figure 4a–c are unannealed, while Figure 4d–f correspond to films annealed at 100 °C. The films demonstrate a long ribbon-like morphology (with lengths greater than 2 mm and widths around 0.5 mm) indicative of long-range molecular ordering at low shearing speeds of 10 and 50 μm/s.

**Figure 4:**
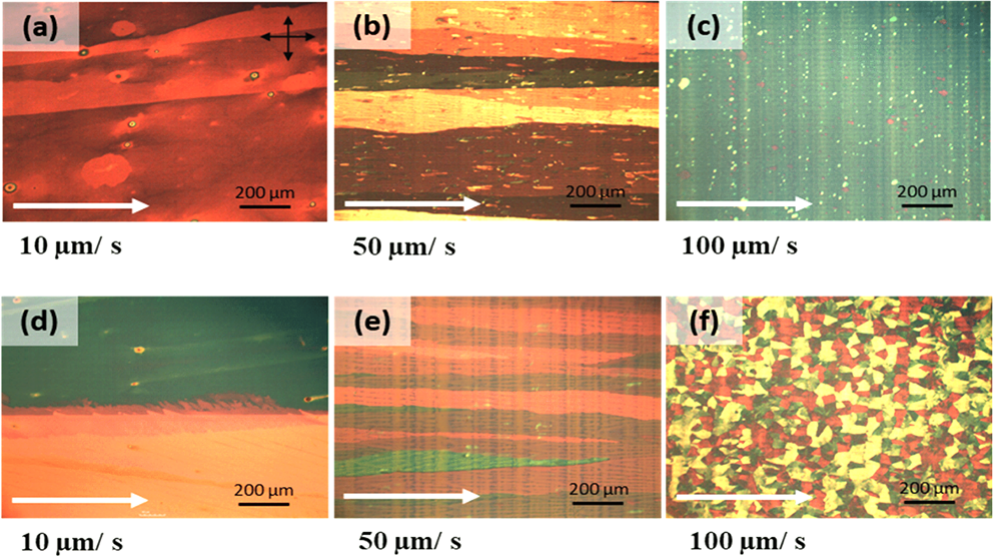
POM images of the DBT-I films deposited from toluene solution, sheared at different shearing speeds on the Si/SiO_2_/PTCS substrate at room temperature. (a–c) Unannealed films and (d–f) annealed films at 100 °C. Shearing speeds: (a, d) 10 μm/s, (b, e) 50 μm/s, and (c, f) 100 μm/s. Scale: 200 μm. The shearing direction is marked with a white arrow.

As the shear speed is increased, both the grain size and ribbon widths decrease. At a speed of 100 μm/s, polycrystalline grains begin to emerge on the films (grains of ∼1 μm size are present in unannealed films, and grains of 50–100 μm size emerge when annealed). Further increasing the shearing speed to 200–500 μm/s leads to a gradual disappearance and dewetting of the films (Figure S4). However, when the shearing speed surpasses 1000 μm/s, the films reappear (Figure S4 g,h). Higher-resolution images of the films at speeds ranging from 200 to 10,000 μm/s can be found in the inset of Figure S4. Additionally, Figure S5 displays POM images of films that were solution-sheared with a substrate temperature of approximately 70 °C. In this case, due to the elevated substrate temperature, films were not formed at lower speeds. Ribbon-like morphologies were obtained at speeds of 50 and 100 μm/s. The presence of more spherical grains was observed at a higher speed of 200 μm/s compared with films coated on substrates at room temperature. These observations vividly illustrate the dependence of the molecular arrangement of DBT-I in films on the temperature and shearing speed.

The variation in film thickness at different shearing speeds is illustrated in Figure S6. The films solution-sheared at speeds ranging from 10 to 30 μm/s exhibit thicknesses in the range of 50–100 nm. A further increase of shearing speed results in a gradual decrease of thickness. For films coated at speeds of 40 and 50 μm/s, the thicknesses are ∼60 nm. However, as the speed increased to 200 μm/s, the film thickness gradually decreased below 10 nm. Due to dewetting, there is almost no film formed until reaching a speed of approximately 1000 μm/s. Nevertheless, at 1000 μm/s, the film reappears with a thickness of approximately 6 nm, and at 10,000 μm/s, a film with a thickness of around 7 nm is obtained. It is important to note that we did not extensively study the thickness variation by depositing films at all speeds. Instead, an average estimation was derived by selecting specific convenient speeds. Hence, the exact speed at which dewetting starts or the films reappear could not be precisely determined. Additionally, slight batch-to-batch variations in speed and thickness were observed, which are also reflected in the subsequent discussion of the device parameters.

The films produced through solution-shearing of DBT-I in chloroform (Figure S7) exhibit characteristics that are distinctly different from those obtained with toluene. When toluene-based films exhibit ribbon-like morphology at low shearing speeds and granular features at higher speeds, the chloroform-based films display granular features at all shearing speeds, with the size of these granules decreasing as the shearing speed increases. Additionally, the morphology of sheared and spin-coated films is similar for chloroform-based films.

In contrast to toluene-based films, proper film formation was not observed at lower shearing speeds due to the high volatility of chloroform. At a shear speed of 100 μm/s, slip-stick films with low wettability are formed. A homogeneous film was achieved at a shearing speed of 500 μm/s. However, as the shearing speed increased to 800 μm/s, the grain size decreased, and issues such as film nonuniformity and reduced wettability reappeared. The impact of substrate heating and the influence of increasing shearing speeds were not extensively analyzed for chloroform-based films due to their fast evaporation and nonhomogeneous film formation. Morphological investigations highlight the substantial influence of solution-processing methods, solvent selection, coating speed, substrate temperature, and annealing conditions on the morphological characteristics of the solution-processed DBT-I films. Furthermore, a comprehensive analysis is conducted to establish the correlation between film morphology and crystallinity and to examine the impact of these factors on the electrical performance of OFETs utilizing DBT-I as the active layer.

### 2.6 AFM Analysis of Thin Films

AFM analysis reveals the surface morphology (Figures S8 and S9) and roughness of thin films (Tables S1 and S2) on a nanometer scale. The unannealed spin coated films from chloroform solution (Figure S8a) exhibit a uniform film with the presence of tiny grains with a root-mean-square (rms) roughness of ∼1.34 nm. When the same film is annealed (Figure S8b), flake-like structures appear with an increased rms roughness of ∼3.4 nm (Table S1). The spin-coated and annealed film from toluene solution (Figure S9a) exhibits more pronounced granular features with island-like surface morphology compared to the chloroform-based film, and the height profile shows a height difference of 20–30 nm (Figure S9a1) between the bottom layer and the raised levels.

The AFM images of solution-sheared films, from toluene, with shearing speeds of 10 and 100 μm/s are shown in Figure S9b,c, respectively. The films exhibit ribbon-like morphology as seen in POM images. A stacking of several ribbon-like structures is also seen in the films with height profiles as shown in Figure S9b1,c1. Apart from that, the film surfaces contain several circular dips, which are more pronounced in the film sheared at 100 μm/s than that at 10 μm/s. Moreover, numerous numbers of spherical structures with heights ranging from 30–40 nm are visible all over the sheared films, which are not visible in the POM images. Although the reason for this feature is not clear, it could be due to the accumulation of solution due to the low shearing speed. We assume that these features may act as charge-trapping sites, causing large hysteresis in solution-sheared films, which will be discussed in the device section.

### 2.7 Thin Film Structural Characterization

On the right side of Figure 3 are the grazing incidence wide-angle X-ray scattering (GIWAXS) patterns of spin-coated films of DBT-I using chloroform and toluene as solvents. GIWAXS patterns were examined for unannealed and annealed films of DBT-I. The unannealed films show a different diffraction pattern than the annealed ones but also reveal a somewhat lower degree of crystalline order—specifically, the out-of-plane peaks are broader than they are after annealing, indicating a lower degree of structural coherence in that direction (Figure 3a,c), whereas highly crystalline films are observed for the annealed films with narrow peaks in both in-plane and out-of-plane directions (Figure 3b,d). More strikingly, the change in peak positions indicates a polymorphic phase transition. Though the broad peaks in the unannealed sample images made indexing more challenging, both unit cells were successfully indexed (Figure S10). **Phase I**, the structure in the as-cast, unannealed samples, has the following unit cell parameters: a = 9.22 Å, b = 5.14 Å, c = 39.8 Å, α = 87.8°, β = 73.0°, γ = 72.5°, whereas the postannealing structure, **phase II**, was indexed according to a = 9.725 Å, b = 15.666 Å, c = 22.143 Å, α = 90.05°, β = 96.59°, and γ = 90.86°. The in situ annealing of DBT-I as shown in Figure S11 also reveals the gradual appearance of **phase II** as the temperature is increased. The molecular orientation of the images at 29 and 50 °C is drastically different from the one at 99 °C, where sharp spots appear, indicating that at this temperature a highly crystalline morphology of **phase II** is reached. This structure is maintained at 149 °C. Further heating to 179 °C destroys the diffraction pattern, attributable to the sample in a molten state. The reappearance of the pattern after cooling down to 98 and then 47 °C indicates recrystallization, mainly in **phase II**, consistent with the DSC data (Figure 2c).

The solution-sheared films exhibit a GIWAXS pattern similar to that obtained for spin-coated films under 360° rotation (removing any degree of in-plane texture). However, unlike spin-coated films, the molecular arrangement and, consequently, the in-plane crystalline texture in solution-sheared films are highly anisotropic. Therefore, GIWAXS patterns were also collected for both parallel and perpendicular shearing directions, as depicted in Figure S12. In the GIWAXS patterns for the parallel and perpendicular directions, highly crystalline features are evident at lower shearing speeds with less-ordered features starting to appear as the shearing speed increases. Also, the diffraction patterns of the film, recorded with the beam parallel and perpendicular to the shearing direction, exhibit clear differences, indicating an in-plane texture within the distribution of crystallites likely induced by the directionality of the solution-shearing procedure. This finding is in agreement with the oriented morphology seen in the corresponding POM images. The differences in patterns become more evident when the shearing speed is low (10 and 50 μm/s, Figure S12a,b,d,e). At a higher shearing speed of 100 μm/s (Figure S12c,f), the GIWAXS patterns of the parallel and perpendicular directions become similar to each other and to the spin-coated films seen in Figure 3. This suggests that at high shearing speeds, the orientation of the crystallites in the films becomes more isotropic, as indicated by the presence of small crystallites of different colors in the POM images of these films. Also, the effect of annealing is not much evident in the GIWAXS patterns of the films sheared at lower speeds (10 and 50 μm/s), whereas it is clearly evident when the shearing speed is 100 μm/s (Figure S12c,f) as found in the case of unannealed and annealed spin-coated films (Figure 3).

### 2.8 OFET Characterization

#### 2.8.1 OFET with Spin-Coated Films of DBT-I

To analyze the correlation between morphology, crystallinity, and electrical performance, OFETs were fabricated using DBT-I films prepared using the various methods discussed in the previous sections. All OFETs in the study were fabricated in the bottom-gate top-contact (BGTC) configuration on Si/SiO_2_ substrates, with or without using SAMs. Figure 5 shows the electrical characteristics of OFETs fabricated on bare Si/SiO_2_ substrates using spin-coated films of DBT-I dissolved in solvents chloroform and toluene. (The output characteristics corresponding to Figure 5 are given in Figure S13.)

**Figure 5:**
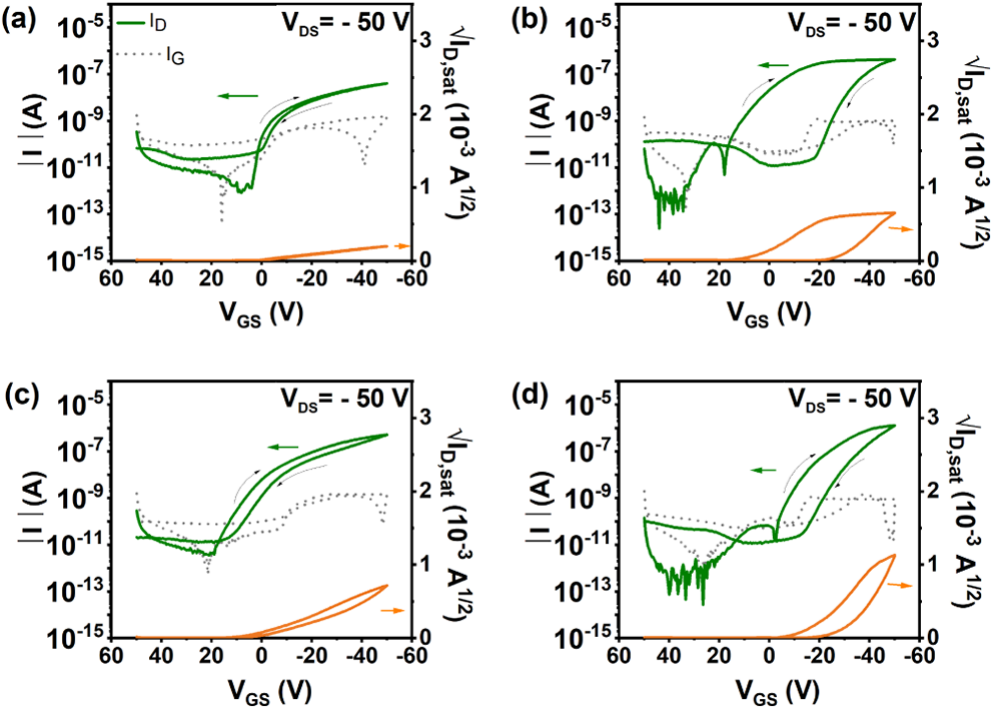
Transfer characteristics of OFETs made with (a, c) unannealed (**phase I**) and (b, d) annealed (**phase II**) films of DBT-I dissolved in (a, b) chloroform and (c, d) toluene, spin-coated on SiO_2_.

It is evident from Figure 5 that the annealed devices exhibit superior electrical performance than the unannealed devices but show a higher amount of hysteresis along with nonideal behavior (nonlinear transfer curve with dual slopes in the forward sweep indicating a gated contact effect) as evident from the transfer curves. To account for the possible overestimation of mobilities due to this nonideal behavior, all the mobilities reported in this work are calculated using a method suggested by H. H. Choi et al.^36^ The films deposited from chloroform show an increase in mobility by 1 order of magnitude when annealed (average mobility without annealing: (1.62 ± 0.64) × 10^–4^ cm^2^ V^–1^ s^–1^ and average mobility with annealing: (1.81 ± 1.64) × 10^–3^ cm^2^ V^–1^ s^–1^). On the other hand, when toluene was used as the solvent, the average mobility almost doubled (average mobility without annealing: (2.14 ± 1.6) × 10^–3^ cm^2^ V^–1^ s^–1^ and average mobility with annealing: (4.84 ± 3.16) × 10^–3^ cm^2^ V^–1^ s^–1^). An overall improved performance was observed when toluene was used as the solvent.

To gain a more comprehensive understanding of the film properties and OFET performance, as well as to mitigate traps responsible for hysteresis in the OFETs, we utilized two SAMs, namely, OTS-18 and PTCS, on the Si/SiO_2_ substrates.

Figures S14 and S15 illustrate the response of OFETs on SAM-coated substrates with chloroform- and toluene-based DBT-I solutions, respectively. The usage of SAMs did not exhibit any significant impact on chloroform-based OFETs. In the case of toluene-based devices, the application of OTS-18 did not improve OFET performance, but the application of PTCS resulted in an enhancement of OFET performance (Figure S15). However, it is important to note that the degree of hysteresis in the transfer characteristics, and with it as the likely underlying cause—the deep trap density—was not reduced with the application of SAMs.

Table 2 summarizes the key figures obtained for the best devices with spin-coated films of DBT-I. These results align with the morphological and crystalline characteristics of the films discussed earlier. A clear correlation between the annealing process, morphology, crystallinity, and electrical performance is observed: when the films are annealed, the grain size increases and the films become more crystalline, resulting in enhanced electrical performance of the devices. However, there is an undesirable degree of hysteresis in these devices’ characteristics, which we believe to be due to the creation of defect states’ consequence of the polymorphic transition. The average figure of merits obtained for five to ten devices in each category are summarized in Table S3, and the average saturation mobility and threshold voltages of OFETs fabricated with spin-coated films of DBT-I under various fabrication conditions are displayed in Figure S16. OFETs with annealed films of DBT-I exhibited a relatively large device-to-device variation, evident from the long error bars. This variation may be associated with the uncontrollable growth of grains, even under similar fabrication conditions. Among all of the fabrication conditions, the OFETs fabricated on PTCS-treated substrates with annealed films of DBT-I dissolved in toluene demonstrated the best performance. They exhibit an average mobility of (5.69 ± 4.40) × 10^–3^ cm^2^ V^–1^ s^–1^ (with the best mobility being 1.47 × 10^–2^ cm^2^ V^–1^ s^–1^) and I_on_/I_off_ of 10^5^. Therefore, further discussions will primarily focus on OFETs fabricated on PTCS-treated substrates, as they have shown the most promising results in terms of performance and mobility.

**Table 2:** Figure of Merits of the Best OFETs Based on Spin-Coated Films of DBT-I^a^

		Threshold voltage, *V*_th_ (V)			
Device specifications (solvent, annealing condition, SAM)	Mobility, μ (cm^2^ V^–1^ s^–1^) (average of forward and backward sweeps)	Forward sweep	Backward sweep	Δ*V*_th_ (V)	$\frac{I_{\mathrm{ON}}}{I_{\mathrm{OFF}}}$
Chloroform, Unannealed	2.57 × 10^–4^	4.44	1.02	3.42	10^4^
Chloroform, Annealed	2.48 × 10^–3^	25.4	–12.2	37.6	10^4^
Chloroform, Annealed, OTS-18	1.34 × 10^–3^	–3.14	–12.8	9.66	10^4^
Chloroform, Annealed, PTCS	2.58 × 10^–3^	5.48	–10.7	16.18	10^4^
Toluene, Unannealed	3.17 × 10^–3^	3.53	–3	6.53	10^4^
Toluene, Annealed	9.14 × 10^–3^	–7.71	–12.4	4.69	10^4^
Toluene, Annealed, OTS-18	1.14 × 10^–3^	–3.14	–10.5	7.36	10^4^
Toluene, Annealed, PTCS	1.47 × 10^–2^	–6.05	–11.9	5.85	10^5^

a Average figure of merits of the OFETs are given in Table S3.

### 2.9 OFETs with Solution-Sheared Films

The performance of OFETs using solution-sheared films of DBT-I was thoroughly examined by using different shearing speeds and thermal treatments. Figure 6 presents the characteristics of OFETs with solution-sheared films of DBT-I (in toluene) at varying shearing speeds on PTCS-coated substrates. The films were deposited at room temperature and subsequently annealed at 100 °C. The characteristics were obtained with the channel parallel to the shearing direction, while the characteristics of the same device with the channel perpendicular to the shearing direction are provided in Figure S17.

**Figure 6:**
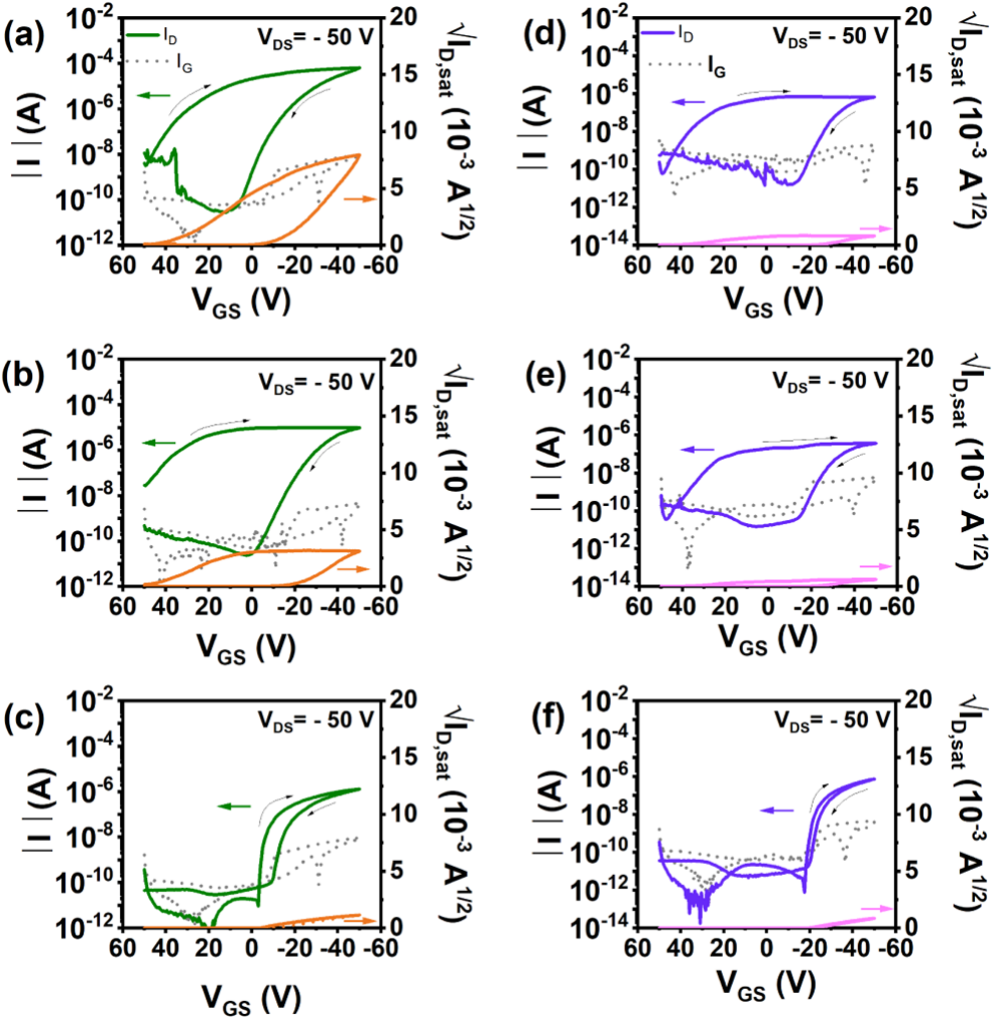
Transfer characteristics of OFETs with solution-sheared films of DBT-I in toluene at different shearing speeds on PTCS. (a, b, c) Characteristics obtained with the channel parallel to the shearing direction and (c, d, e) characteristics obtained with the channel perpendicular to the shearing direction. Shearing speeds: (a, d) 10 μm/s, (b, e) 50 μm/s, and (c, f) 100 μm/s. The films were deposited at room temperature and annealed at 100 °C.

In this study, devices with the channel parallel to the shearing direction exhibited significantly higher mobility compared to those with the channel perpendicular to the shearing direction, particularly when the film had a ribbon-like morphology. The dependence of the direction on OFET performance was further evaluated for a film solution-sheared at 10 μm/s using a round mask with channels at different angles, as illustrated in Figure S18. Previous studies have also reported the impact of shearing direction on the crystallization direction and molecular orientation of the films.^37–40^ The calculation of charge carrier mobility anisotropy between parallel and perpendicular shear directions revealed an anisotropy ratio μ∥/μ⊥ of 216. This value is significantly higher than the anisotropy ratios that were previously reported for thin films and organic single crystals.^41–44^ Notably, the charge mobility anisotropy findings align with the GIWAXS data presented in Figure S12. This could indicate an intrinsic high degree of anisotropy in the packing of the molecules after the polymorphic transition in conjunction with the morphological anisotropy due to the solution-shearing. Given that the ratio is high because one of the directions exhibits very low mobility, it could also stem from thermally induced effects like stress fractures in the highly textured, crystalline films whose presence—even though we do not have evidence for them from AFM—could significantly reduce the mobility in one direction.

In the case of solution-sheared devices, another trend is observed: as the shearing speed increases, the performance of the OFETs decreases. Upon comparing the film morphology at different speeds, it becomes apparent that at lower speeds (10 and 50 μm/s), the film adopts a ribbon-like morphology with a considerably larger grain size and has a highly crystalline nature. However, as the speed increases, the grain size decreases (above 100 μm/s), leading to an increase in grain boundary density and, consequently, defect density. Consistent with the findings in spin-coated devices, films with higher grain sizes and high crystallinity exhibit better OFET performance in terms of maximum current and field-effect mobility. Unfortunately, these high-performing devices with large grains are associated with relatively large hysteresis. This may be attributed to the fact that as grain size increases, charge transport improves due to enhanced crystallinity and long-range order within the grains. However, simultaneously, the film thickness also increases as the shearing speed decreases (as seen in Figure S6), potentially contributing to the creation of bulk traps, resulting in significant hysteresis. For instance, films solution-sheared at speeds of 10, 50, and 100 μm/s have thicknesses of approximately 50, 60, and 15 nm, respectively, while the thickness of spin-coated films is around 15 nm. A clear correlation among film thickness, morphology, device performance, and the presence of hysteresis emerges here. Thick DBT-I films, formed by solution-shearing at speeds ≤50 μm/s, contain large ribbon-like grains with higher crystallinity, resulting in OFETs with higher on-current values and mobilities but significant hysteresis, whereas thin DBT-I films, formed by spin-coating or solution-shearing at speeds greater than 50 μm/s, show granular features with lower crystallinity, likely leading to low-performing OFETs with negligible hysteresis. If not for thickness, the above observation of the occurrence of high degrees of hysteresis in highly crystalline films with fewer grain boundaries and lower degrees of hysteresis in less ordered films with numerous grain boundaries would contradict the general observation of a large number of traps associated with a higher amount of grain boundaries in films exhibiting granular features.^45–47^

Notably, OFETs with solution-sheared films, particularly when the film morphology is ribbon-like, display significant contact resistance, as evidenced by the S-shaped (nonohmic) output curves (Figure 6a,c), indicating the presence of large Schottky barriers. In contrast, the devices with films of grain-like morphology (spin-coated or solution-sheared) exhibited less contact effects (Figures S13 a–d and 6e). It is worth mentioning that thicker films would also have a larger access resistance, further contributing to contact resistance.^48,49^ Here, the solution-sheared films at lower speeds are a lot thicker than the other films, and hence, their contact effects are higher. Moreover, the threshold voltage of devices with large contact effects is also higher.^50^ The distinct morphological features of solution-sheared films, such as the large spherical structures on the surface and stacked layer-like morphology, may have contributed for the increased contact effects.

Figure S19a,b shows the transfer characteristics of OFETs with solution-sheared and unannealed films of DBT-I on PTCS at room temperature, with shearing speeds of 10 and 100 μm/s, respectively. Despite being unannealed, the devices coated at 10 μm/s demonstrate superior performance and a clear presence of large hysteresis (likely due to a high number of traps). In contrast, at a shearing speed of 100 μm/s, the transfer characteristics exhibit lower performance but are relatively hysteresis-free. Interestingly, the OFET performance with unannealed and annealed solution-sheared films exhibits almost comparable characteristics. Whereas, when the films are deposited on PTCS with a substrate temperature of 70 °C, the device mobility is enhanced, in the case of OFETs with films solution-sheared from chloroform-based solution, the electrical performance at different speeds is roughly the same, but a difference is present between annealed and unannealed devices, as shown in Figure S20.

Table 3 provides a summary of the electrical parameters obtained for the best devices under different solution-shearing conditions using toluene as the solvent. As mentioned previously, the mobilities are calculated using the Podzorov method and averaged for forward and backward sweeps to accommodate the influence of hysteresis. The best electrical performance was obtained for the OFET with the DBT-I layer sheared at a speed of 10 μm/s, which exhibited a mobility of 0.26 cm^2^ V^–1^ s^–1^ and a current on–off ratio of 10^6^. Remarkably, these results were consistent regardless of the film’s annealing conditions. Tables S4 and S5 summarize the average device parameters of the OFETs fabricated with shear-coated films of DBT-I with toluene and chloroform as solvents, respectively. Figure S21 depicts the average saturation mobility and average threshold voltages obtained for solution-sheared films in the parallel and perpendicular directions for both toluene- and chloroform-based devices.

**Table 3:** Figure of Merits of the Best OFETs Fabricated with Shear-Coated Films of DBT-I with Toluene as Solvent^a^

			*V*_th_ (V)			
Speed (μm/s)	Direction of shearing	Mobility (cm^2^ V^–1^ s^–1^) (average of forward and backward sweeps)	Forward sweep	Backward sweep	Δ*V*_th_ (V)	*I*_on_/*I*_off_
No substrate temperature, no annealing						
10	∥	2.47 × 10^–1^	35.9	– 27.9	63.8	3.54 × 10^6^
	⊥	8.80 × 10^–3^	61.2	– 27	88.2	2.8 × 10^5^
100	∥	1.05 × 10^–3^	6.27	–2.72	8.99	2.2 × 10^5^
	⊥	8.58 × 10^–4^	4.58	– 1.75	6.33	1.3 × 10^5^
No substrate temperature, annealed at 100 °C						
10	∥	2.68 × 10^–1^	34.6	– 17.2	51.8	2.36 × 10^6^
	⊥	2.30 × 10^–3^	40.7	– 23.8	64.5	2.82 × 10^4^
50	∥	3.47 × 10^–2^	45.3	–10.3	55.6	3.9 × 10^5^
	⊥	1.65 × 10^–2^	35.8	– 10.7	46.5	2.27 × 10^4^
100	∥	9.29 × 10^–3^	0.92	–10.7	11.62	2.86 × 10^4^
	⊥	1.47 × 10^–3^	–19.8	– 20.6	0.8	1.38 × 10^5^
Substrate temperature of 70 °C, annealed at 100 °C						
50	∥	7.46 × 10^–2^	6.88	– 18.8	25.68	1.22 × 10^6^
	⊥	9.36 × 10^–4^	14.4	– 25.2	39.6	9.05 × 10^3^
100	∥	2.34 × 10^–2^	15.9	–24.4	40.3	3.58 × 10^5^
	⊥	3.57 × 10^–4^	35.4	– 27	62.4	7.20 × 10^3^
200	∥	5.59 × 10^–3^	4.5	–16.5	21	1.93 × 10^4^
	⊥	2.85 × 10^–3^	2.68	– 17.5	20.18	4.06 × 10^4^

a Average figure of merits of toluene- and chloroform-based solution-sheared devices are given in Tables S4 and S5, respectively.

## 3 Conclusion

In the present work, the synthesis, detailed analysis, and application of the novel DPP-based small molecule DBT-I are presented. By introducing solubilizing alkyl chains at α,ω-positions and short *n*-propyl at N-positions of the DPP core, the molecular planarity and intermolecular interactions are maximized. DBT-I effectively produces uniform thin-films with both spin-coating and solution-shearing techniques. However, the film morphology, crystallinity, and device performance show significant differences, investigated in detail by using different processing parameters, surface analysis techniques, and device fabrication. Solution shearing is found to be an effective way of producing highly crystalline DBT-I thin films, which offer high OFET performance in comparison with spin-coated films. Moreover, the degree of crystallinity was found to be highly dependent on the solution-shearing speed and direction. A direct correlation between the morphology and OFET performance was also found. It was observed that larger grain sizes fostered better overall device performance, though they presented higher hysteresis. These phenomena were observed for both spin- and solution-sheared devices. The semiconductor morphology plays a critical role in determining the OFET characteristics. We found that a rational variation of fabrication parameters, such as solvent, deposition method, shearing speed and direction, and annealing, could lead to distinct DBT-I morphologies and molecular arrangements, which in turn enable the optimization of OFET performance.

## 4 Experimental Section

### 4.1 Synthesis of DBT-I

Under nitrogen atmosphere, DPP-Br_2_ (272.2 mg, 0.502 mmol, 1 equiv) and T-Sn (582.5 mg, 1.10 mmol, 2.2 equiv) were dissolved in 20 mL of chlorobenzene and put into a preheated bath at 130 °C. A full solution was achieved after 10 min, and then, the catalytic system was added, comprised of Pd_2_dba_3_ (9.19 mg, 0.01 mmol, 2% mol equiv) and tri­(ortho-tolyl)­phosphine (12.2 mg, 0.04 mmol, 8% mol equiv) previously dissolved in 1 mL of chlorobenzene in an inert atmosphere. After 4.25 h of reaction, the flask was removed from the oil bath, and then, the reaction mixture was diluted with 40 mL of CHCl_3_ and stirred for 10 min. The mixture was poured into 10% HCl. The organic fraction was separated and washed with water and brine, then dried over MgSO_4_, filtered, and evaporated. The crude product was purified by flash chromatography using hexane and DCM as eluents. The final product was obtained as a dark blue film in 59.6% yield.

^1^H NMR (CDCl_3_, 500 MHz) δ [ppm] = 8.92 (d, 2H, J= 4.1 Hz), 7.25 (d, 2H, J= 4.1 Hz), 7.17 (d, 2H, J= 3.5 Hz), 6.72 (d, 2H, J= 3.5 Hz), 4.07 (t, 4H, J= 7.6 Hz), 2.76 (d, 4H, J= 6.6 Hz), 1.82 (sxt, 4H, J= 7.5 Hz), 1.66 (br s, 2H), 1.31 (br m, 64H), 1.05 (t, 6H, J= 7.4 Hz), 0.89 (t, 12H, J= 6.9 Hz).

### 4.2 Nuclear Magnetic Resonance (^1^H NMR)

NMR spectra were recorded on a Bruker Avance III 500 spectrometer operating at 500.13 MHz for ^1^H by using a 5 mm ^1^H/^13^C/^19^F/^31^P gradient probe. The samples were measured in CDCl_3_ at room temperature. The spectra were referenced to the residual solvent peak.

### 4.3 UV–vis Spectroscopy

UV–vis absorbance in solution was measured on a Cary 6000i (Varian) spectrometer with an integration time of 0.3 s and a spectral bandwidth of 1 nm. Fluorescence was determined using a Fluorolog 3 (Horiba Jobin Yvon, USA) at excitation wavelengths of 316 and 360 nm with integration times of 0.2 s and spectral bandwidths of 1 nm. The samples were dissolved at a concentration of 0.05 mg/mL. For solid-state measurements, a Cary 5000 UV–vis–NIR (Agilent Technologies) at a scan rate of 20 nm·s^–1^ was used. Polymer thin films were prepared by drop casting or spin coating on quartz substrates.

### 4.4 Cyclic Voltammetry (CV)

Measurements were performed using a nitrogen-bubbled solution of 0.1 M tetra-*n*-butylammonium hexafluorophosphate (*n*-Bu_4_NPF_6_) in acetonitrile, recorded at a scan rate of 50 mV/s, and referenced to the ferrocene/ferrocenium (Fc/Fc^+^) redox couple. The reference electrode consisted of a silver wire in acetonitrile containing 0.1 M *n*-Bu_4_NPF_6_ and 0.01 M AgNO_3_. The counter electrode consisted of a platinum wire, and the working electrode consisted of a platinum disk. Polymer samples were prepared as films deposited on top of the working electrode by drop-casting from a CHCl_3_ solution.

### 4.5 Thermal Analysis

TGA was performed on a TGA Q500 of TA Instruments with a heating rate of 10 °C/min under nitrogen using Pt crucibles and 10 mg of sample for each analysis. DSC was carried out with a DSC2500 of TA Instruments (Waters) in the temperature range of −60–300 °C under a nitrogen atmosphere at a scan rate of 10 °C/min. All samples were investigated in a heating–cooling–heating cycle.

### 4.6 Thin Film Fabrication

Thin films of DBT-I were deposited on either pristine SiO_2_ substrates following ultraviolet-ozone (UVO) treatment for 15 min or on SAM-coated SiO_2_ substrates as per requirement. The SAMs, PTCS, and OTS-18 were deposited by following the procedure reported elsewhere.^51–53^ DBT-I, dissolved in either chloroform or toluene at a concentration of 5 mg/mL was deposited using spin-coating (1000 rpm for 1 min) or the solution-shearing method (at different speeds) as described in the main text. The films were used without or with annealing (at 100 °C) as required by the experiment. Also, substrate heating was opted as per requirement during solution-shearing.

### 4.7 Polarized Optical Microscopy

Microscopic images of the films were taken by using an optical microscope (MOTIC BA310Met) with a crossed polarizer.

### 4.8 Atomic Force Microscopy

Surface morphology of thin films was obtained using a Nanosurf atomic force microscope in tapping mode with TAP-190Al-G nonconducting tips from Budget Sensors.

### 4.9 GIWAXS

The GIWAXS images in Figure 3a,b were recorded at the NCD-SWEET beamline of the ALBA synchrotron. An area detector (LX255-HS, Rayonix) was placed approximately 18 cm behind the sample. The beam size was 130 μm horizontally and 30 μm vertically, and the beam energy was 12.4 keV. The incidence angle was 0.10°, and the exposure time was 1 s. The collected images were calibrated by using a chromium oxide calibration standard. The images in Figure S8 used almost the same settings, but with a sample–detector distance of 16.7 cm, 150 μm horizontal and 70 μm vertical beam sizes, and exposure times of 0.5 s.

The images shown in Figure 3c,d were recorded at the Sirius beamline at the SOLEIL synchrotron in Paris, France. A Pilatus 1 M detector was placed approximately 38 cm behind the sample. The beam size was 500 μm horizontally and 70 μm vertically, and the beam energy was 12 keV. The incidence angle was 0.10°, and the exposure time was 150 s. The collected images were calibrated by using a silver behenate calibration standard.

The in situ heating images shown in Figure S11 were recorded at the ID10 beamline at the ESRF synchrotron in Grenoble, France. A Pilatus 300K detector was used. The beam size was 40 μm horizontally and 40 μm vertically, and the beam energy was 10 keV. The incidence angle was 0.14°, and the exposure time was 2 s.

The GIWAXS data were corrected and analyzed with WxDiff.

### 4.10 OFET Fabrication and Characterization

The OFETs were fabricated in the bottom gate-top contact (BGTC) configuration on highly n-doped silicon wafers with a 300 nm SiO_2_ layer, where Si acts as the gate electrode and SiO_2_ acts as the gate dielectric. The semiconductor channel material DBT-I was deposited on the substrate as described in the previous section. In some of the devices, prior to semiconductor deposition, SAM layers such as the OTS and PTCS were applied following the procedure mentioned above. Source and drain electrodes were deposited by the thermal evaporation of gold (∼80 nm) using shadow masks of different channel lengths (100 μm, 125 μm) and width (3 mm).

Electrical characterizations of the OFETs were performed by using a Keysight B1500 Semiconductor Device Parameter Analyzer. The mobility in the saturation regime (μ_sat_) was calculated using the following equation:

1 $$\mu_{sat}=\frac{2L}{WC_{i}}{\left(\frac{\partial \sqrt{I_{DS}}}{\partial V_{GS}}\right)}^{2}$$

where *C*_i_ is the capacitance per unit area of the gate dielectric (11 nF cm^–2^), *W* is the channel width, and *L* is the channel length.

## Supplementary Material



## Notes

Additional experimental details such as material synthesis and characterization, film morphologies (POM, AFM), GIWAXS, device fabrication methods, electrical characteristics of devices, and tables of electrical parameters (PDF)
