# Supplementary material for: Morphology, Crystallinity, and Electrical Performance of Solution-Processed OFETs Based on a DPP-Based Small Molecule
Source: ACS Appl Electron Mater. 2025 Jul 15;7(15):6874–84. doi: 10.1021/acsaelm.5c00731 (PMC12356105; doi:10.1021/acsaelm.5c00731)
Supplement: Supplementary file 1 [file el5c00731_si_001.pdf]

Supporting Information

Morphology, Crystallinity and Electrical Performance of Solution-Processed OFETs based on a DPP-based Small Molecule

Enrique Caldera-Cruz [†, a, e], Reshma Raveendran [†, b], Yevhen Karpov [c], Felix Talnack [b], Brigitte Voit [a, e], Anton Kiriy [d], Stefan C. B. Mannsfeld [*, b]

[a] *Leibniz-Institute of Polymer Research Dresden, Hohe Straße 6, 01069 Dresden, Germany*
[b] *Center for Advancing Electronics Dresden and Faculty of Electrical and Computer Engineering, Technische Universität Dresden, Helmholtzstraße 18, 01069 Dresden, Germany*
[c] *Novaled GmbH Dresden, Elisabeth-Boer-Strasse 9, 01099 Dresden, Germany*
[d] *beeOLED GmbH, Niedersedlitzer Strasse 75c, 01257 Dresden, Germany*
[e] *Organic Chemistry of Polymers, Technische Universität Dresden, 01062 Dresden, Germany*

*E-mail: stefan.mannsfeld@tu-dresden.de

† E.C. and R.R. contributed equally to this work

Synthesis

DPP-nPr. Under argon atmosphere, DPP-H (2 g, 6.66 mmol, 1 eq.,) was charged into a two-neck flask equipped with a condenser, along with K_2CO_3 (3.68g, 36.64 mmol, 5.5 eq.) and 50 ml of dry DMF. The mixture was stirred for 1 hour at 100 °C. Afterwards, n-bromopropane (14.65 mmol, 2.2 eq.) was added and the reaction was kept at 50°C overnight. Subsequently the solvent was evaporated, the raw product was extracted with DCM, washed with water, and dried over $MgSO_4$. The product was purified by column chromatography with DCM as eluent, obtained at 59% yield.

^{1}H-NMR (CDCl3, 500 MHz) δ [ppm] = 8.92 (dd, 2H, J= 3.8, 0.9 Hz), 7.64 (dd, 2H, J= 5.0, 1.3 Hz), 7.29 (dd, 2H, J= 4.9, 3.9 Hz), 4.05 (t, 4H, J= 7.6 Hz), 1.79 (sxt, 4H, J= 7.6 Hz), 1.02 (t, 6H, J= 7.4 Hz).

DPP-Br$_2$. Under nitrogen atmosphere and light protection, 17 ml of a $CHCl_3$ solution of NBS (0.34 g, 1.92 mmol, 2.1 equivalent) was added dropwise over 15 minutes at 0 °C to DPP-nPr (0.35 g, 0.912 mmol, 1 equivalent), previously dissolved in 41 ml of anhydrous $CHCl_3$. Then, the mixture was stirred over 48 hours at room temperature. Afterwards it was extracted and washed with water, dried over MgSO4, and the solvent was evaporated under reduced pressure. The purification was done by flash chromatography using DCM as eluent and recrystallized from CHCl3. the final product was a dark solid, obtained at 56.7 % yield.

^{1}H-NMR (CDCl3, 500 MHz) δ [ppm] = 8.69 (d, 2H, J= 4.4 Hz), 7.25 (d, 2H, J= 4.1 Hz), 3.98 (t, 2H, J= 7.9 Hz), 1.77 (sxt, 4H, J= 7.5 Hz), 1.02 (td, 6H, J= 7.4, 3.5 Hz).

T-C$_{20}$. A 250 ml round bottom flask charged with thiophene (5.0 g, 59.4 mmol, 1 eq.) was flushed with argon and 100 ml of dry THF were added. The flask was cooled down to -78 °C and 25 ml of n-BuLi (2.5 M in hexane, 62.4 mmol, 1.05 eq.) were added slowly. After 1 hour, 9-(iodomethyl)nonadecane was added dropwise (25.5 g, 62.4 mmol, 1.05 eq.) and the reaction was left stirring overnight at room temperature. The reaction was subsequently quenched with 120 ml of water and extracted with diethyl ether. The raw product was purified by column chromatography with petroleum ether as eluent. Yield: 55.1 %

^{1}H-NMR (CDCl3, 500 MHz) δ [ppm] = 7.11 (d, 1H, J= 4.7 Hz), 6.92 (dd, 1H, J= 4.7, 3.5 Hz), 6.76 (d, 1H, J= 2.8 Hz), 2.77 (d, 2H, J= 6.6 Hz), 1.63 (br s, 1H), 1.27 (br s, 32H), 0.90 (t, 6H, J= 6.9 Hz).

T-Sn. Under argon atmosphere, T-C20 (1.82 g, 5 mmol, 1 eq.) was dissolved in 20 ml of dry THF. Then, n-BuLi (2.5 M in hexane, 2.1 ml, 5.25 mmol, 1.05 eq.) were added dropwise -78 °C and the reaction mixture was stirred for 1 h. Solution of trimethyltin chloride (1.0 M in THF,

5.25 mmol, 1.05 eq.) was added dropwise into the reaction mixture and left stirring overnight. The next day the reaction mixture was extracted with diethyl ether, washed with water, and dried over $MgSO_4$. The raw product was used without further purification in the next reactions. Mass of the crude material 2,25 g.

^{1}H-NMR (CDCl3, 500 MHz) δ [ppm] = 7.02 (d, 1H, J= 3.2 Hz), 6.88 (d, 1H, J= 3.2 Hz), 2.80 (d, 1H, J= 6.6 Hz), 1.64 (br s, 1H), 1.28 (br s, 32H), 0.90 (t, 6H, J= 6.9 Hz), 0.35 (s, 9H).

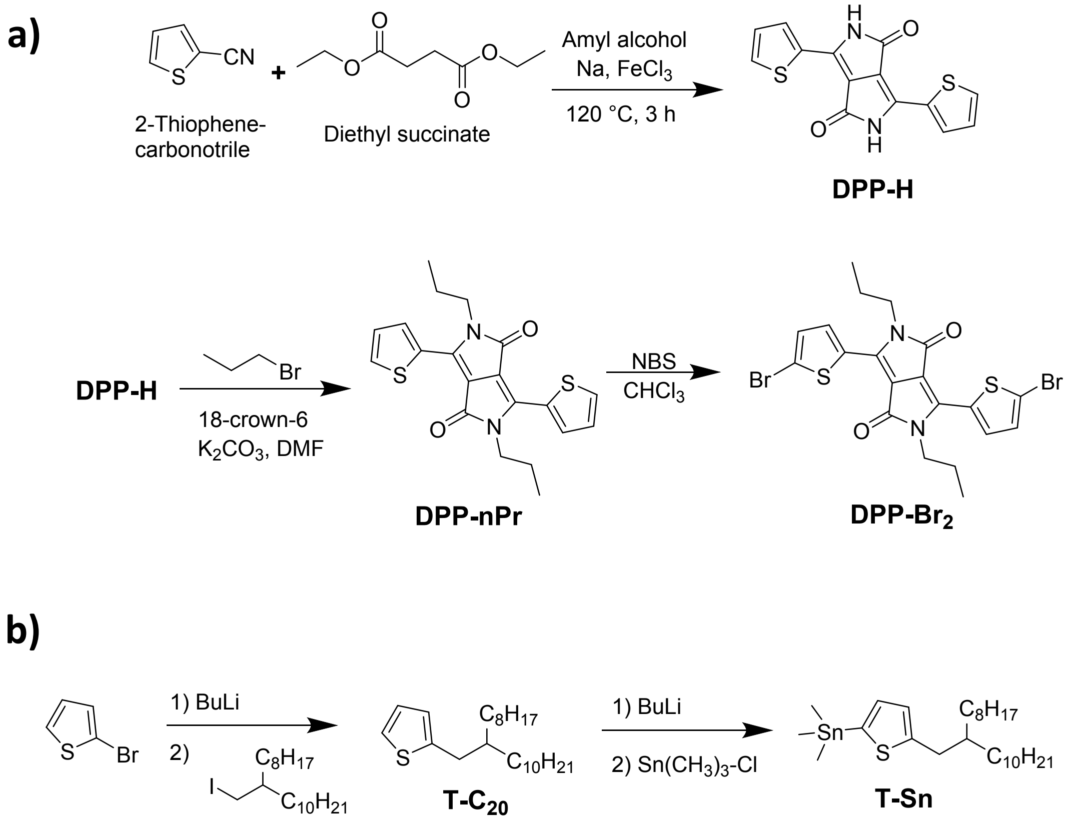

c)

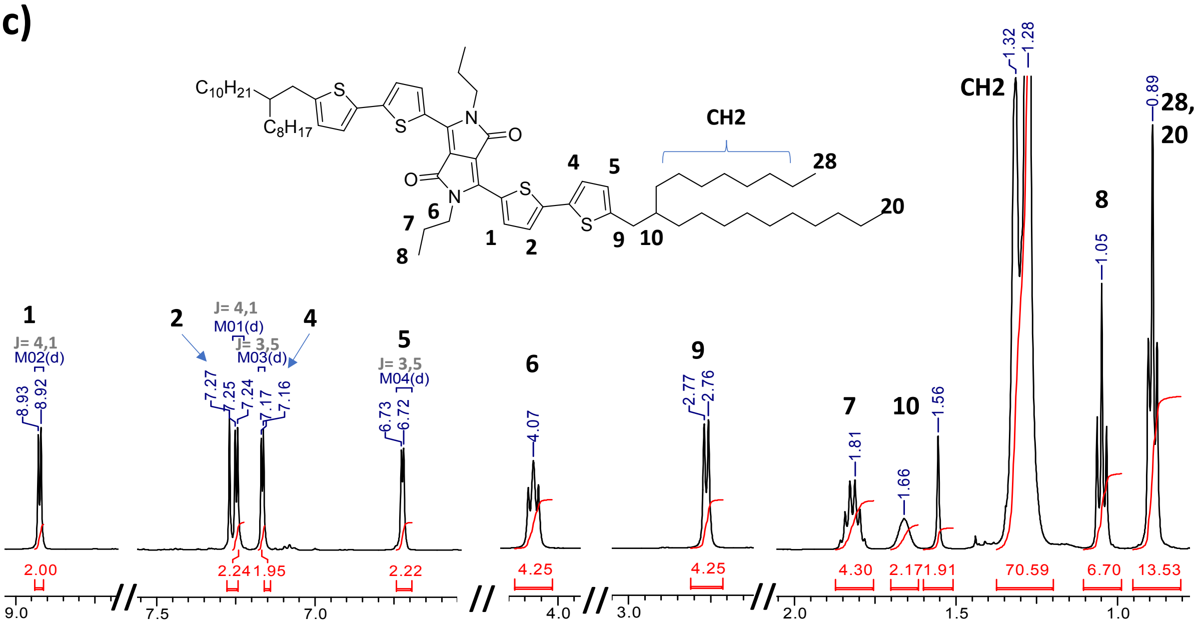

d)

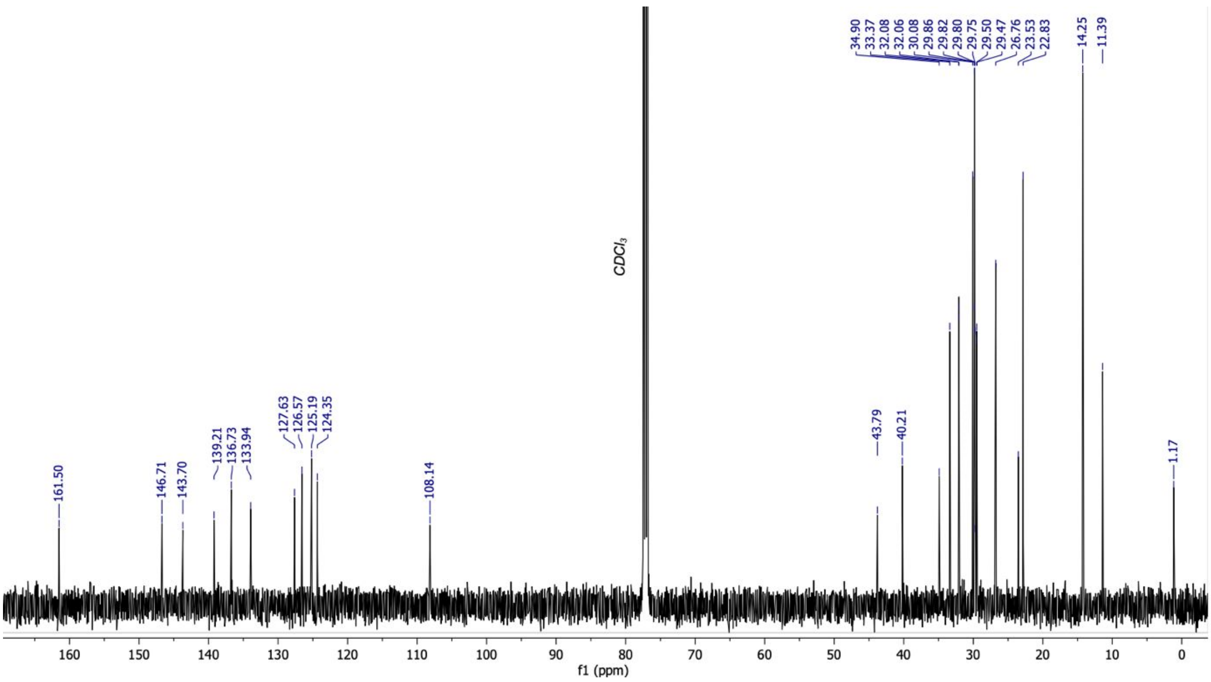

Figure S1. Synthetic route of the precursors of DBT-I. (a) DPP core synthesis; (b) thiophene functionalization. (c) ^{1}H-NMR and (d) ^{13}C-NMR spectra of DBT-I recorded in $CDCl_3$.

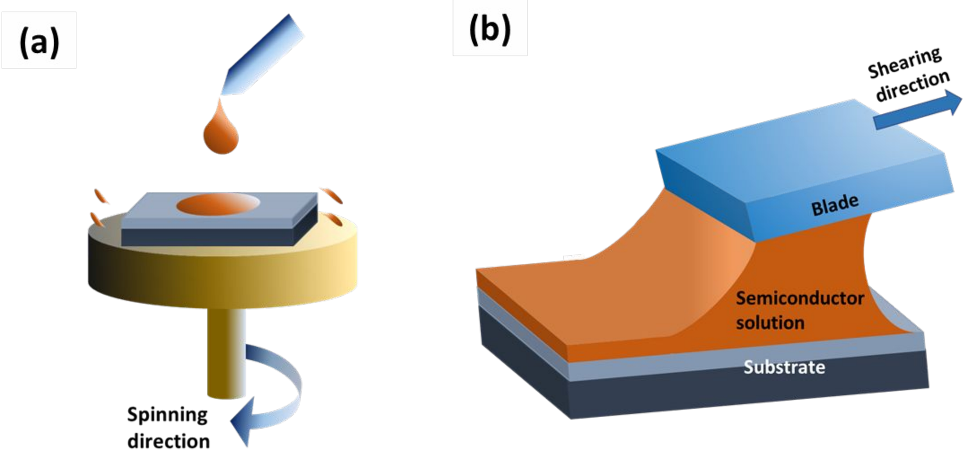

Figure S2. Schematic representation of solution-processing using (a) spin-coating and (b) solution-shearing methods.

Spin-coating and solution-shearing: In the spin-coating method, a small quantity of solution is dispensed onto a substrate and rapidly rotated at a high angular velocity, facilitating uniform spreading of the solution and gradual solvent evaporation, resulting in the formation of a thin film **(Fig. S2a).**[54] Conversely, in the meniscus-guided solution-shearing method, a small droplet of solution is deposited on one end of a flat substrate and then dragged to the opposite end using a blade moving at a constant speed **(Fig. S2b).**[54–56] Furthermore, in the solution-shearing method, the substrate can be heated during film deposition. Both spin and solution-shearing techniques possess their own advantages and disadvantages. Spin-coating, while producing highly uniform films with precise thickness control, tends to result in significant solution wastage. On the other hand, solution- shearing requires only a small amount of solution with minimal loss. However, solution-shearing encounters challenges related to reproducibility and thickness variations.[51]

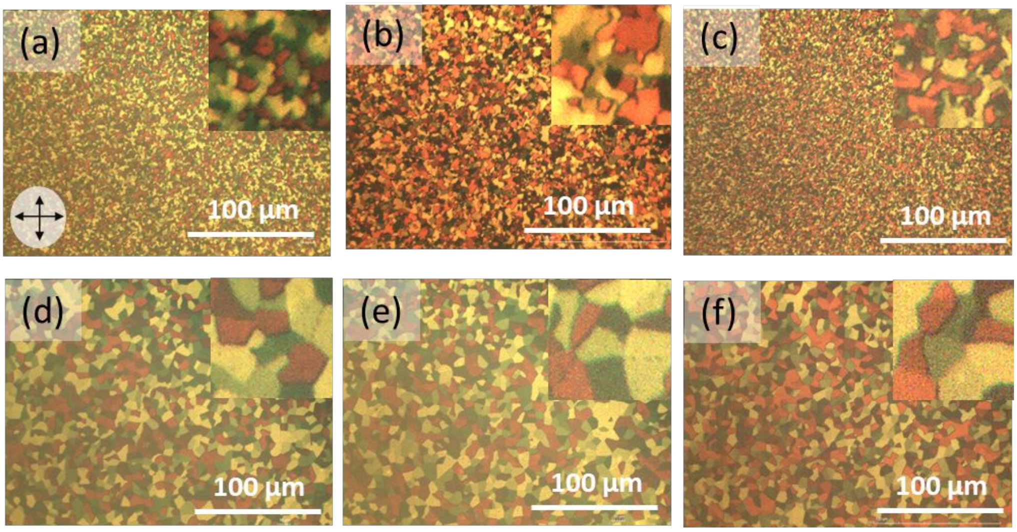

Figure S3. POM images of the DBT-I film dissolved in (a-c) chloroform and (d-f) toluene, and deposited on (a, d) SiO_2, (b, e) OTS-18 and (c, f) PTCS.

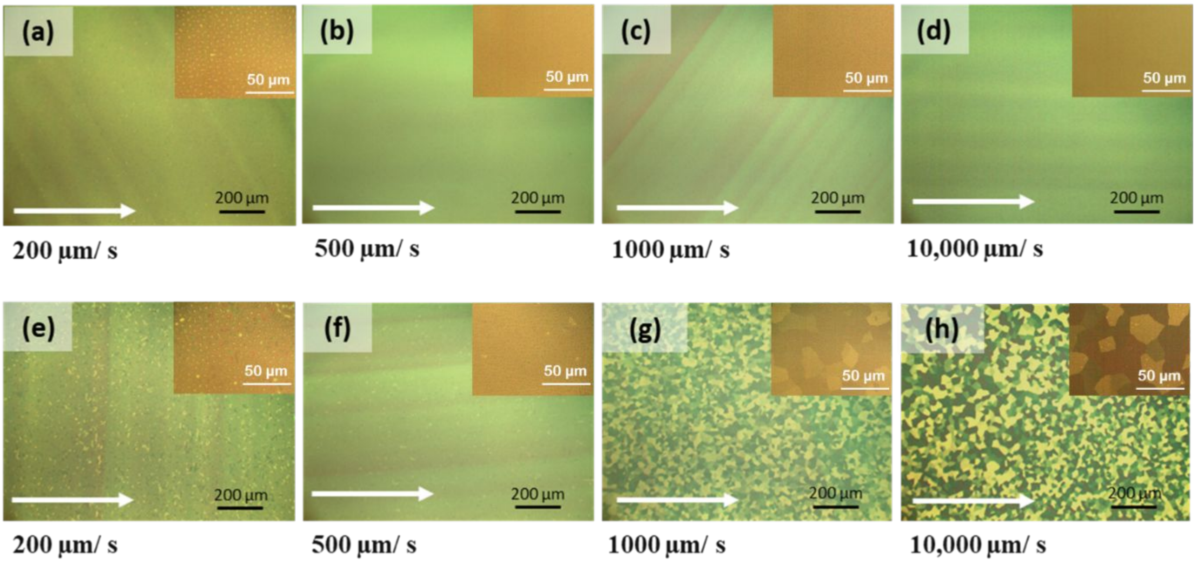

Figure S4. POM images of the DBT-I films deposited from toluene solution, sheared at higher shearing speeds on Si/SiO_2/ PTCS substrate at room temperature. (a-d) Un-annealed films and (e-h) annealed films at 100 °C. Shearing speeds: (a, e) 200 µm/s, (b, f) 500 µm/s, (c, g) 1000 µm/s, (d, h) 10,000 µm/s. Scale: 200 µm. Shearing direction is marked with a white arrow. Inset shows high resolution images of the same.

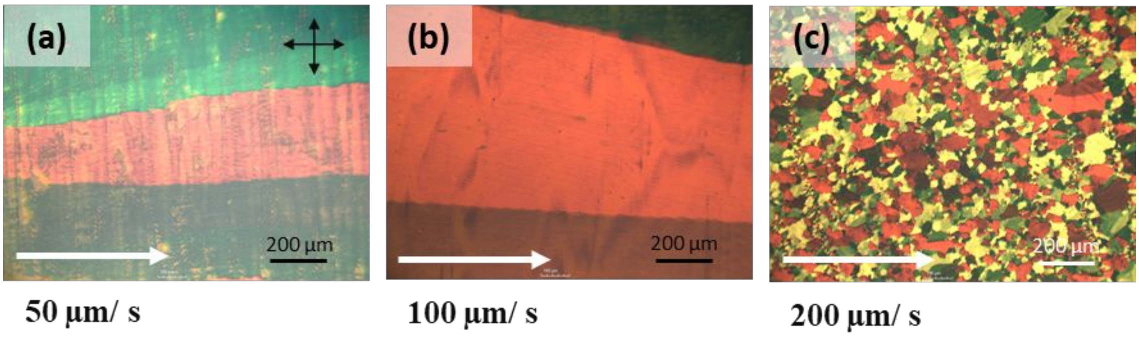

Figure S5. POM images of DBT-I films solution-sheared on PTCS at a substrate temperature of 70 ^{0}C with shear speeds (a) 50 µm/s, (b) 100 µm/s and (c) 200 µm/s, and annealed at 100 ^{0}C. Scale: 200 µm. Shearing direction is marked with white arrow. Solvent: toluene

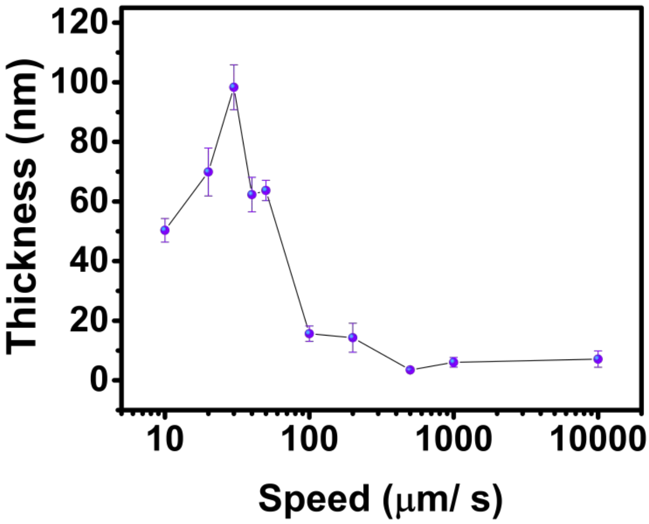

Figure S6. Plot showing the variation of film thickness with shearing speed. The films are made from DBT-I dissolved in toluene and are solution-sheared on PTCS substrate at room temperature.

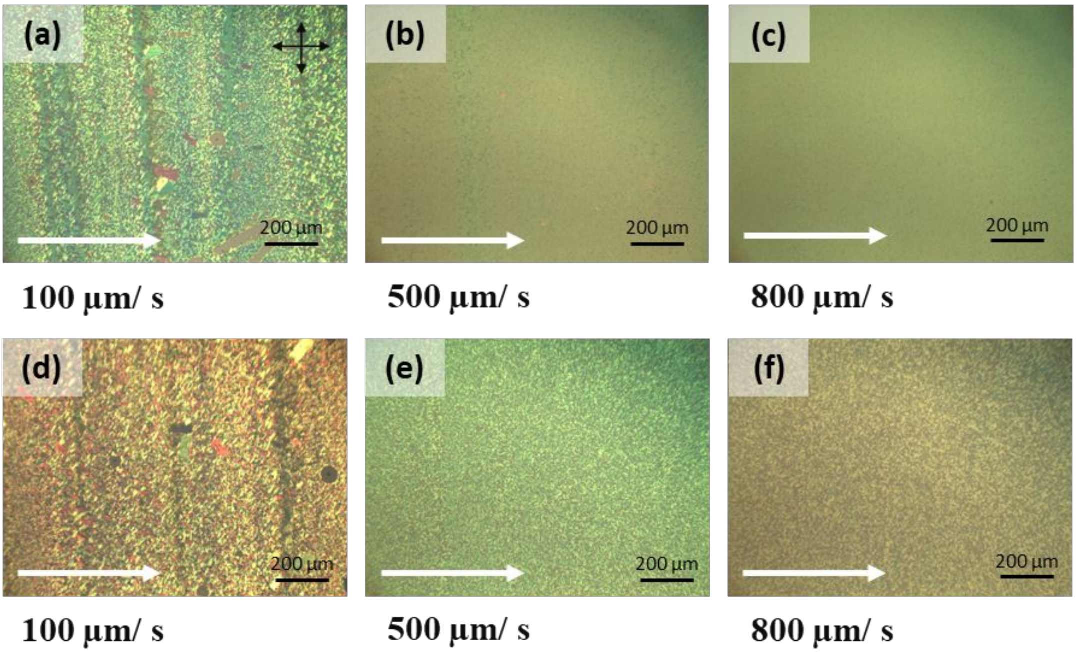

Figure S7. POM images of the shear coated films of DBT-I in chloroform at different shearing speeds on PTCS, without substrate temperature. (a-c) un-annealed films and (d- f) annealed (at 100 ^{0}C) films coated using shear speeds of (a, d) 100 µm/s, (b, e) 500 µm/s and (c, f) 800 µm/s. Scale: 200 µm. Shearing direction is marked with white arrow.

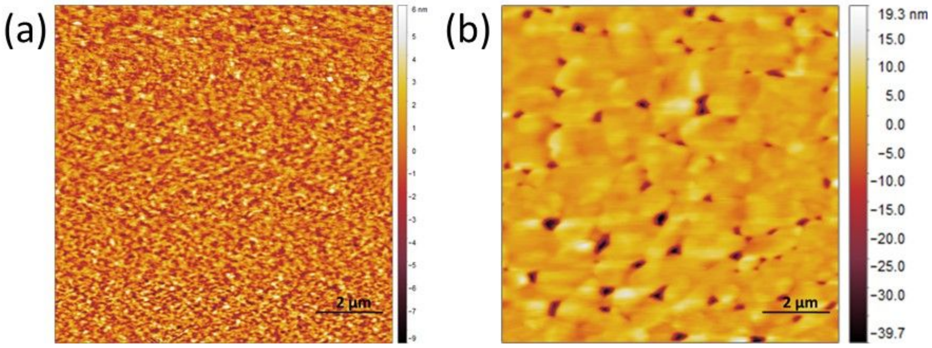

Figure S8: AFM images of spin coated films of DBT-1 from chloroform solution (a) unannealed, (b) annealed at 100 ^{0}C.

Table S1. Roughness of spin coated films shown in figure S8.

**Unannealed film**	**Annealed film**
1.34 nm	3.40 nm

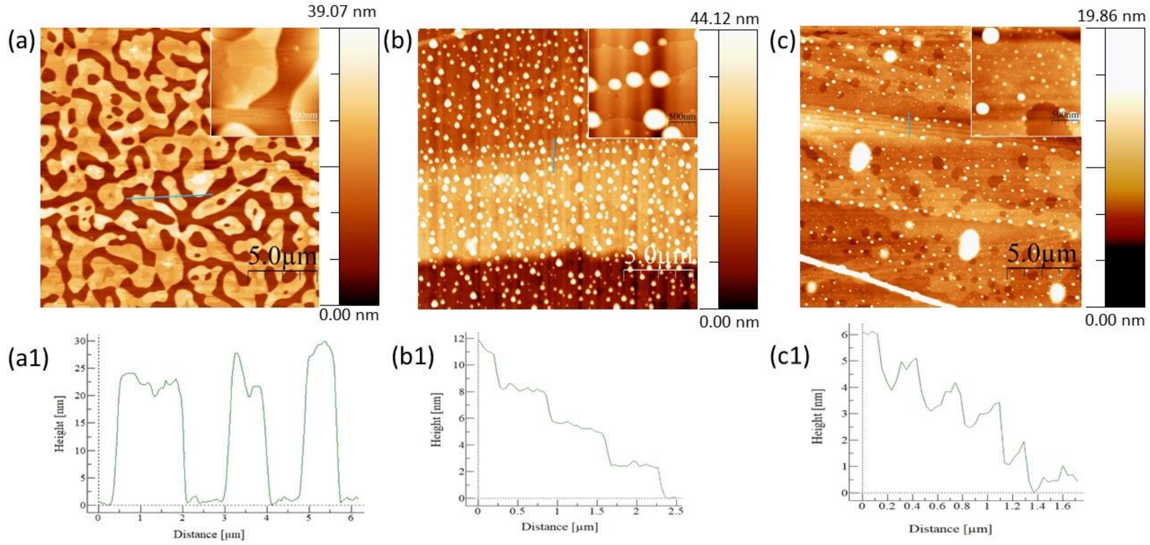

Figure S9: AFM images of (a) spin coated and (b, c) solution sheared films (direction: left to right) of DBT-1 from toluene solution. The shearing speeds are (b) 10 µm/s and (c) 100 µm/s. All films are annealed at 100 ^{0}C. (a1, b1, c1) Height profiles of selected region from each film. Inset shows zoomed portions from each film.

Table S2. Roughness of spin coated films shown in figure S9.

**(a) Spin coated film**	**b) Film solution sheared at 10 um/s**	**(c) Film solution sheared at 100 um/s**
9.6 nm	10 nm	2.96 nm

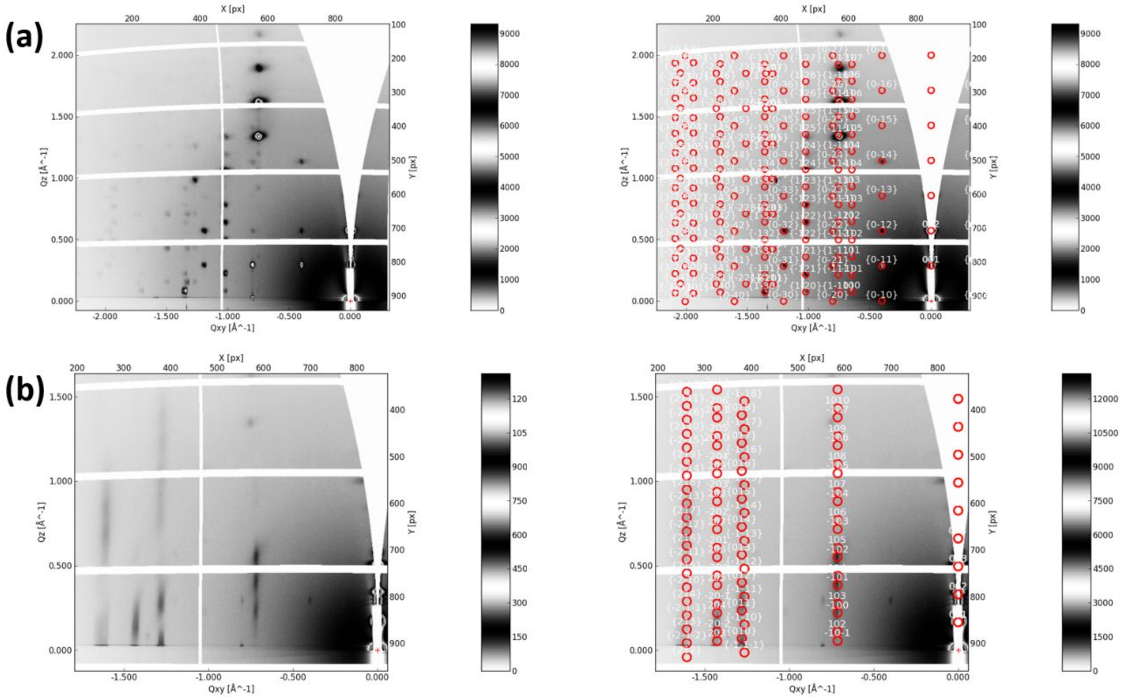

Figure S10. Indexed unit cells of (a) annealed and (b) unannealed films of DBT-I deposited from toluene- based solution.

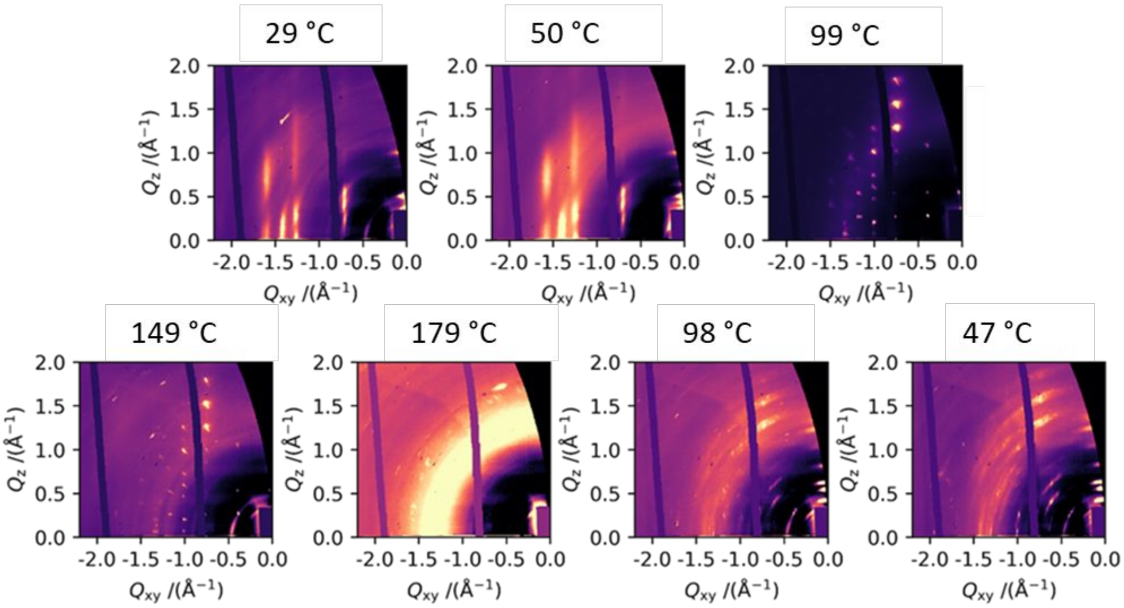

Figure S11. GIWAXS patterns of the spin-coated DBT-I films under different in-situ annealing temperatures.

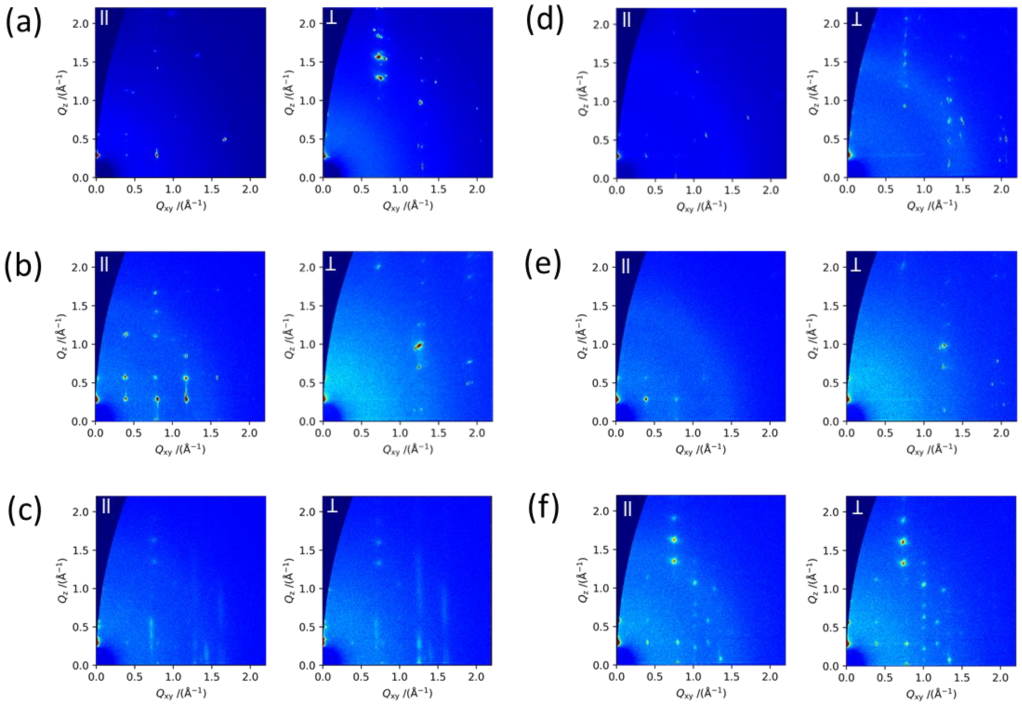

Figure S12. GIWAXS patterns of the shear coated DBT-I films (in toluene) in the parallel and perpendicular shearing directions at speeds of (a, d)10, (b, e) 50 and (c, f) 100 µm/ s. The films are coated on PTCS with no substrate heating among which the films (a-c) are unannealed and (d-f) are annealed at 100 ^{0}C.

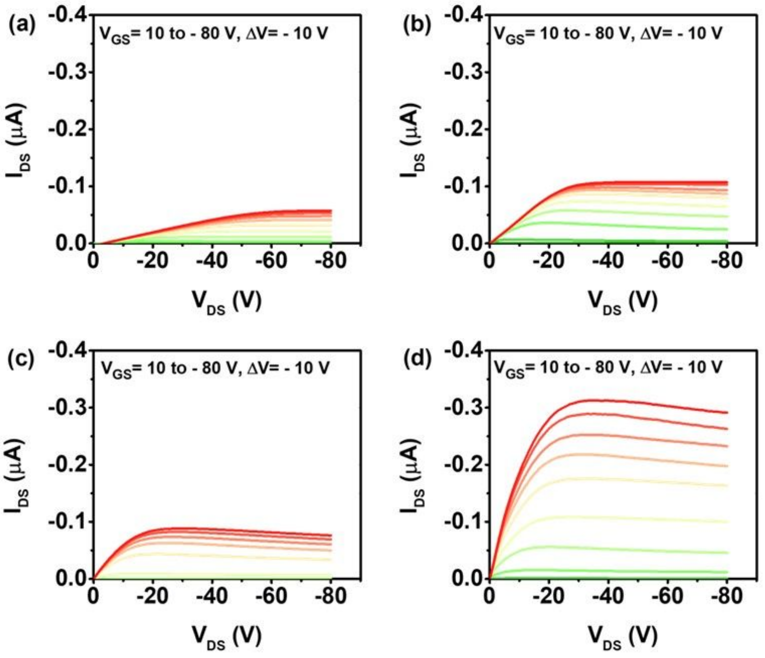

Figure S13. Output characteristics of OFETs made with (a, c) un-annealed (**phase I**) and (b, d) annealed (**phase II**) films of DBT-I dissolved in (a, b) chloroform and (c, d) toluene, spin-coated on SiO_2.

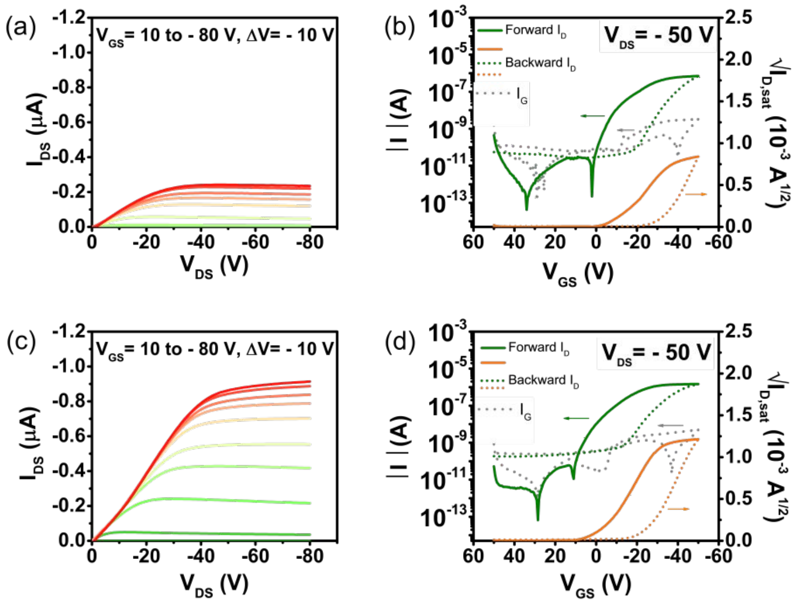

Figure S14. Electrical response of OFETs made with spin- coated DBT-I on different substrates. (a, c) Output and (b, d) transfer characteristics of OFETs fabricated on (a, b) OTS-18 treated, and (c, d) PTCS treated SiO_2. The devices were made with DBT-I dissolved in chloroform.

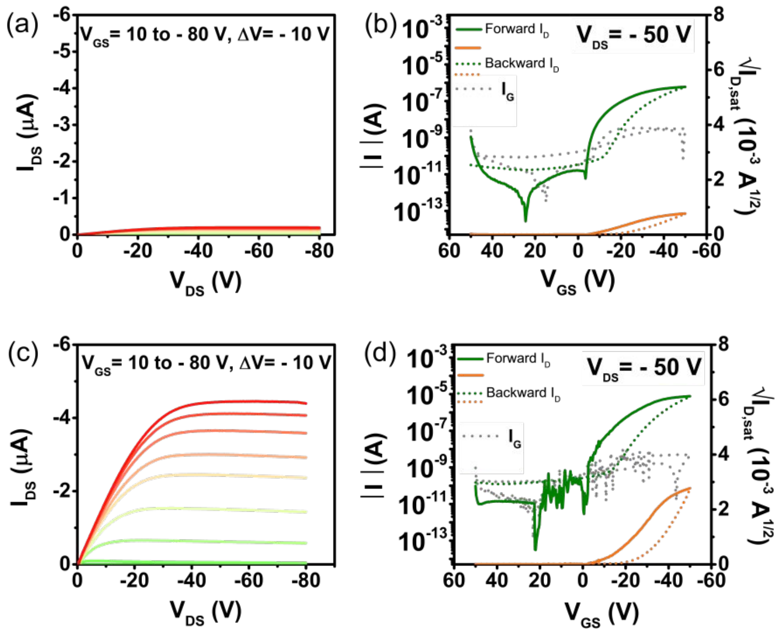

Figure S15. Electrical response of OFETs made with spin- coated DBT-I on different substrates. (a, c) Output and (b, d) transfer characteristics of OFETs fabricated on (a, b) OTS-18 treated, and (c, d) PTCS treated SiO_2. The devices were made with DBT-I dissolved in toluene.

Table S3. Average figure of merits of OFETs based on spin-coated films of DBT-I.

**Device specifications (Solvent, annealing condition, SAM)**	**Mobility, μ (cm^2 V^{-1} s^{-1})**			**Threshold voltage, V_{th} (V)**		**I_{On}/ I_{off}**
	Forward sweep	Backward sweep	Average	Forward sweep	Backward sweep	
Chloroform, Unannealed	(1.68 ± 0.60) x 10^{-4}	(1.55 ± 0.64) x 10^{-4}	(1.62 ± 0.64) x 10^{-4}	2.26 ± 4.46	-3.26 ± 9.4	10^4
Chloroform, Annealed	(1.56 ± 1.35) x 10^{-3}	(2.06 ± 1.75) x 10^{-3}	(1.81 ± 1.64) x 10^{-3}	-6.85 ± 16.73	-29.77 ± 12.3	10^4
Chloroform, Annealed, OTS-18	(2.21 ± 1.84) x 10^{-3}	(1.69 ± 0.62) x 10^{-3}	(1.95 ± 1.45) x 10^{-3}	-5.4 ± 4.08	-22.15 ± 8.11	10^4
Chloroform, Unannealed, PTCS	(1.65 ± 1.33) x 10^{-5}	(1.77 ± 1.27) x 10^{-5}	(1.71 ± 1.22) x 10^{-5}	12.65 ± 2.19	12.33 ± 1.29	10^3

Chloroform, Annealed, PTCS	$(1.64 \pm 1.49) \times 10^{-3}$	$(3.13 \pm 1.24) \times 10^{-3}$	$(2.39 \pm 1.53) \times 10^{-3}$	9.11 ± 15.36	-20.44 ± 2.23	10^4
Toluene, Unannealed	$(2.06 \pm 1.52) \times 10^{-3}$	$(2.22 \pm 1.79) \times 10^{-3}$	$(2.14 \pm 1.6) \times 10^{-3}$	-6.91 ± 14.04	-18.59 ± 13.74	10^4
Toluene, Annealed	$(4.86 \pm 3.22) \times 10^{-3}$	$(4.82 \pm 3.24) \times 10^{-3}$	$(4.84 \pm 3.16) \times 10^{-3}$	-10.74 ± 4.87	-21.68 ± 7	10^4
Toluene, Annealed, PTCS	$(5.76 \pm 4.59) \times 10^{-3}$	$(5.63 \pm 4.52) \times 10^{-3}$	$(5.69 \pm 4.40) \times 10^{-3}$	-5.8 ± 3.6	-13 ± 7.49	10^5
Toluene, Annealed, OTS-18	$(2.28 \pm 1.00) \times 10^{-3}$	$(2.39 \pm 1.09) \times 10^{-3}$	$(2.34 \pm 0.94) \times 10^{-3}$	-6.4 ± 0.39	-20.54 ± 5.68	10^4

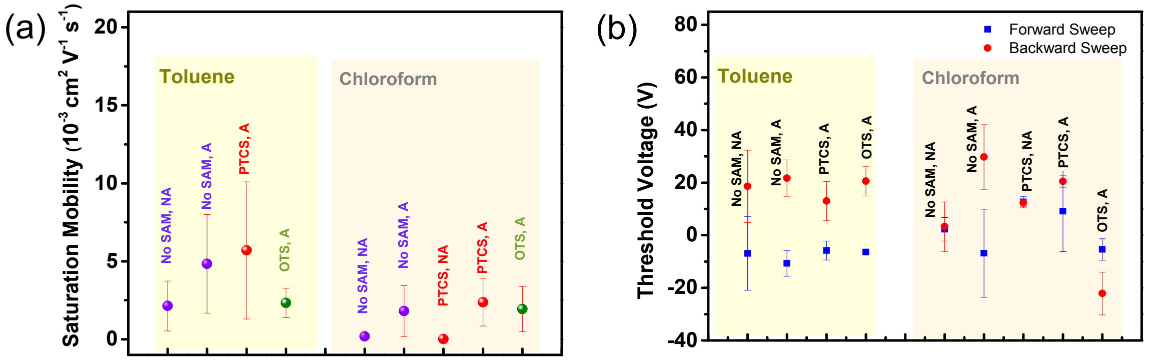

Figure S16. Plots showing (a) the average saturation mobility and (b) threshold voltage of OFETs fabricated with spin coated, annealed (A) or unannealed (NA) films of DBT-I dissolved in toluene or chloroform, coated at a spin speed of 1000 rpm, with or without using SAM.

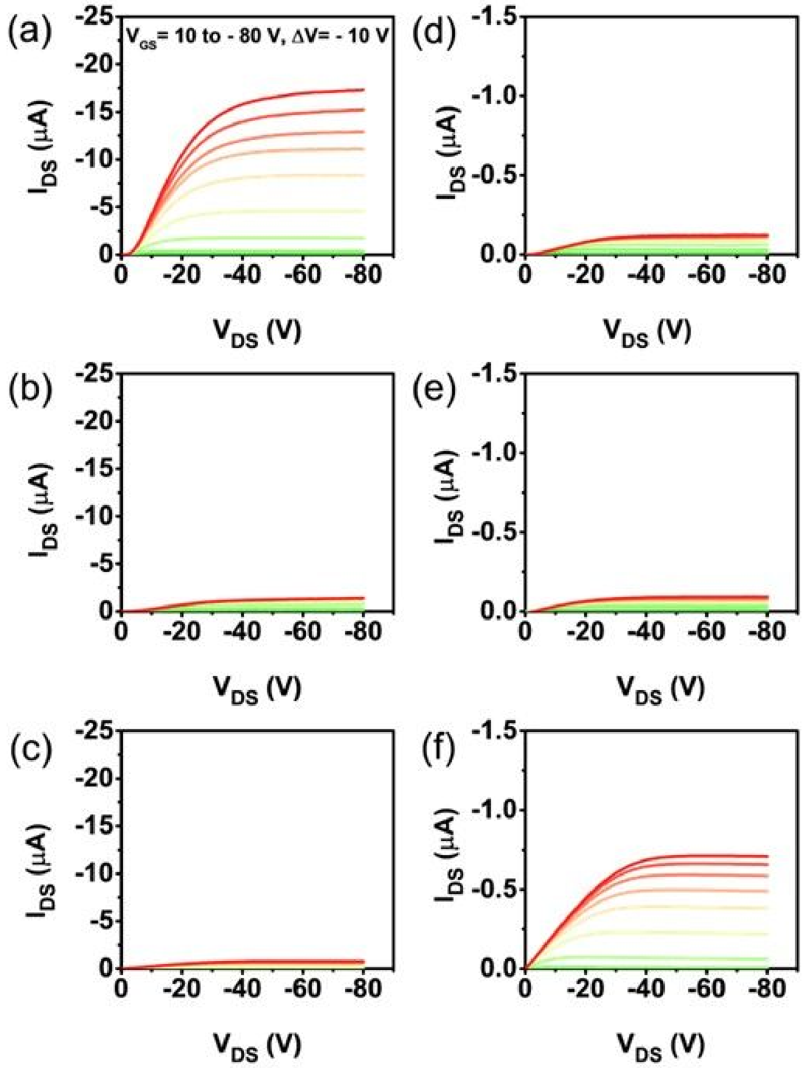

Figure S17. Output characteristics of OFETs with solution-sheared films of DBT-I in toluene at different shearing speeds on PTCS. (a, b, c) characteristics obtained with channel parallel to the shearing direction, (c, d, e) characteristics obtained with channel perpendicular to the shearing direction. Shearing speeds: (a, d) 10 μm/s, (b, e) 50 μm/s and (c, f) 100 μm/s. The films were deposited at room temperature and annealed at 100 °C.

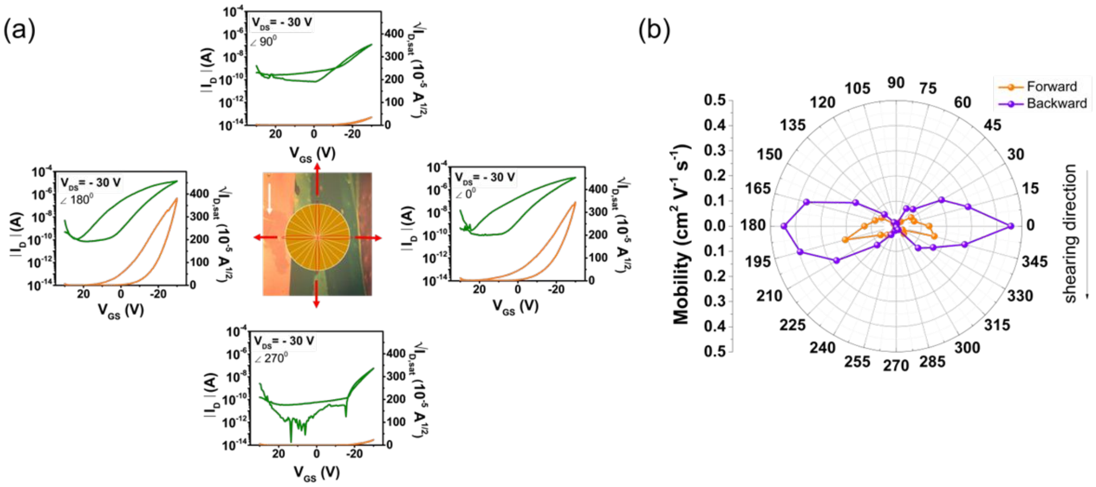

Figure S18. Schematic showing the variation of OFET performance with respect to channel direction. (a) Plots showing the transfer characteristics when the channel is (i, iii) parallel or (ii, iv) perpendicular to the shearing direction. The shearing direction of the film is represented using a white arrow. (b) Polar plot showing the saturation mobilities of the OFETs in (a). The shearing speed is 10 μ m/ s. A circular mask containing 24 channels (L = 125 μm, W= 3 mm) with 15^0 gaps is used.

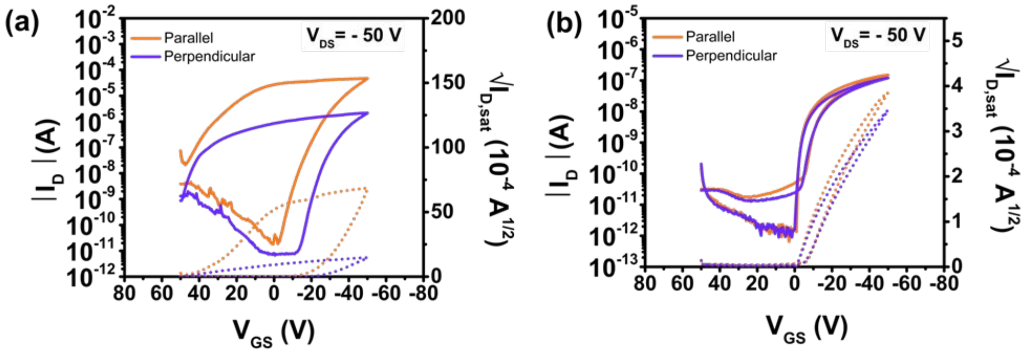

Figure S19. Transfer characteristics of OFETs in the parallel and perpendicular shearing direction using DBT-I (in toluene) at shearing speeds of (a) 10 μm/s and (b) 100 μm/s. The films are deposited at room temperature on PTCS and are not annealed.

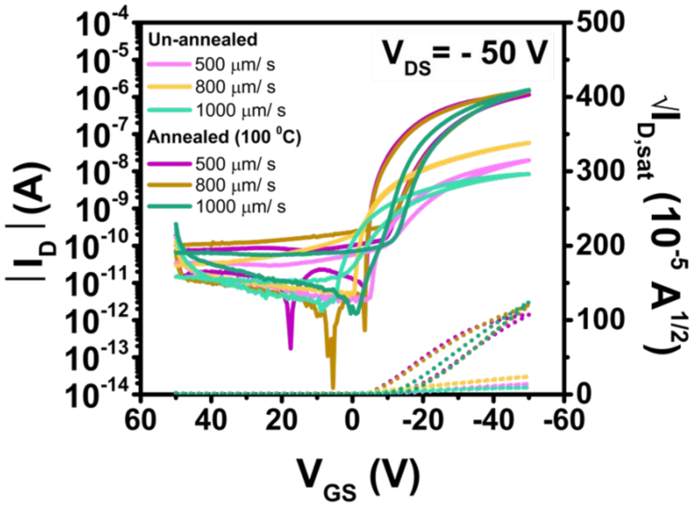

Figure S20. Transfer characteristics of OFETs in the parallel shearing direction using annealed and unannealed films of DBT-I (in chloroform) at shearing speeds of 500, 800 and 1000 µm/s. The films are deposited at room temperature on PTCS.

Table S4. Figure of merits of the OFETs fabricated with shear coated films of DBT-I with toluene as solvent.

**Speed (µm/ s)**	**Direction of shearing**	**Mobility ($cm^2 V^{-1} s^{-1}$)**			**V_{th} (V)**		**I_{on}/ I_{off}**
		Forward sweep	Backward sweep	average	Forward sweep	Backward sweep	
On SiO_2, No substrate temperature, not annealed							
100	||	(1.73 ± 0.74) x 10^{-3}	(1.73 ± 0.74) x 10^{-3}	(1.73 ± 0.68) x 10^{-3}	-7.54 ± 3.18	-8.12 ± 3.17	10^4
	⊥	(9.75 ± 1.10) x 10^{-4}	(9.70 ± 1.09) x 10^{-4}	(9.73 ± 1.03) x 10^{-4}	-10.6 ± 1.17	-11.1 ± 1.28	10^2
On SiO_2, No substrate temperature, annealed							
50	||	(1.20 ± 1.07) x 10^{-2}	(1.25 ± 1.10) x 10^{-2}	(1.22 ± 1.04) x 10^{-2}	-14.9 ± 7.06	-21.30 ± 8.99	10^6
	⊥	(2.43 ± 0.97) x 10^{-3}	(2.42 ± 0.99) x 10^{-3}	(2.43 ± 0.90) x 10^{-3}	-14.3 ± 3.81	-23.80 ± 7.06	10^5
100	||	(2.32 ± 0.65) x 10^{-3}	(2.28 ± 0.62) x 10^{-3}	(2.30 ± 0.61) x 10^{-3}	-7.18 ± 3.78	-18.50 ± 6.12	10^5
	⊥	(4.77 ± 2.06) x 10^{-3}	(4.69 ± 2.06) x 10^{-3}	(4.73 ± 1.99) x 10^{-3}	-7.20 ± 2.34	-14.2 ± 4.38	10^5
On PTCS/ SiO_2, No substrate temperature, not annealed							
10	||	(1.41 ± 1.10) x 10^{-1}	(2.65 ± 1.20) x 10^{-1}	(2.02 ± 1.26) x 10^{-1}	10.9 ± 19.16	-16.2 ± 9.26	10^6

	⊥	(2.21 ± 2.03) x 10^{-2}	(2.63 ± 2.23) x 10^{-2}	(2.42 ± 2.09) x 10^{-2}	-0.58 ± 3.3	-6.27 ± 1.72	10^5
100	||	(9.95 ± 1.93) x 10^{-4}	(9.35 ± 1.74) x 10^{-4}	(9.65 ± 1.8) x 10^{-4}	-2.26 ±2.32	-4.49 ± 2.79	10^5
	⊥	---	---	---	---	---	---
On PTCS, No substrate temperature, annealed at 100 ^{0}C							
10	||	(9.31± 8.78) x 10^{-2}	(2.19± 1.81) x 10^{-1}	(1.56 ± 1.54) x 10^{-1}	9.52 ± 23.76	-15 ± 7.98	10^6
	⊥	(6.31± 5.77) x 10^{-3}	(9.03± 5.35) x 10^{-3}	(7.67 ± 5.65) x 10^{-3}	7.16 ± 23.16	-19.5 ± 6.88	10^4
50	||	(2.38± 1.95) x 10^{-2}	(4.91± 1.78) x 10^{-2}	(3.65 ± 2.19) x 10^{-2}	25.5 ± 23.18	-11.6 ± 3.89	10^5
	⊥	(2.95 ± 2.40) x 10^{-3}	(1.39 ± 1.36) x 10^{-2}	(8.40 ± 12.2) x 10^{-3}	26.7 ± 20.97	-19.5 ± 6.04	10^4
100	||	(5.63 ± 2.99) x 10^{-3}	(5.58 ± 2.94) x 10^{-3}	(5.60 ± 2.84) x 10^{-3}	-9.18 ± 5.71	-14.4 ± 5.2	10^4
	⊥	(6.36 ± 4.38) x 10^{-3}	(6.31 ± 4.41) x 10^{-3}	(6.34 ± 4.19) x 10^{-3}	-9.5 ± 6.92	-13.8 ± 5.96	10^5
On PTCS, Substrate temperature of 70 ^{0}C, annealed at 100 ^{0}C							
50	||	(4.81 ± 4.18) x 10^{-2}	(8.15 ± 7.07) x 10^{-2}	(6.48 ± 5.97) x 10^{-2}	1.97 ± 10.7	-9.22 ± 2.8	10^6
	⊥	(1.39 ± 1.22) x 10^{-3}	(1.49 ± 1.27) x 10^{-3}	(1.44 ± 1.2) x 10^{-3}	-14.4 ± 2.19	- 17.9 ± 1.66	10^3
100	||	(1.29 ± 0.50) x 10^{-2}	(2.24 ± 2.02) x 10^{-2}	(1.77 ± 1.55) x 10^{-2}	-2.96 ± 5.69	-13.2 ± 4.14	10^5
	⊥	(4.80 ± 3.91) x 10^{-4}	(5.01 ± 3.91) x 10^{-4}	(4.90 ± 3.68) x 10^{-4}	-10.6 ± 4.46	-15.1 ± 3.99	10^3
200	||	(3.20 ± 1.91) x 10^{-3}	(3.32 ± 1.95) x 10^{-3}	(3.26 ± 1.84) x 10^{-3}	-7.98 ± 6.77	-14.3 ± 4.86	10^4
	⊥	---	---	---			---

Table S5. Figure of merits of the OFETs fabricated with shear coated films of DBT-I with chloroform as the solvent.

Speed (µm/ s)	Direction of shearing	Mobility ($cm^2 V^{-1} s^{-1}$)			V_{th} (V)		I_{on}/ I_{off}
		Forward sweep	Backward sweep	average	Forward sweep	Backward sweep	
No substrate temperature, not annealed							
500	||	(1.55 ± 1.47) x 10^{-4}	(1.46 ± 1.39) x 10^{-4}	(1.50 ± 1.34) x 10^{-4}	-4.38 ± 3.07	-11.7 ± 2.29	10^4
	⊥	(9.65 ± 3.13) x 10^{-5}	(8.74 ± 3.22) x 10^{-5}	(9.19 ± 2.64) x 10^{-5}	-4.70 ± 5.40	-10.3 ± 7.39	10^4

800	||	(2.46 ± 1.44) x 10^{-5}	(2.14 ± 1.10) x 10^{-5}	(2.30 ± 1.23) x 10^{-5}	-0.84 ± 2.79	-1.51 ± 3.96	10^4
	⊥	(8.38 ± 2.25) x 10^{-5}	(7.87 ± 2.18) x 10^{-5}	(8.12 ± 2.10) x 10^{-5}	-5.79 ± 5.89	-7.29 ± 5.33	10^4
1000	||	(3.98 ± 3.25) x 10^{-4}	(4.01 ± 3.25) x 10^{-4}	(4.00 ± 3.06) x 10^{-4}	3.21 ± 0.85	0.65 ± 1.00	10^4
	⊥	(4.63 ± 0.05) x 10^{-4}	(4.60 ± 0.08) x 10^{-4}	(4.62 ± 0.06) x 10^{-4}	2.91 ± 1.20	1.25 ± 1.13	10^4
No substrate temperature, annealed at 100 ^{0}C							
500	||	(8.08± 1.15) x 10^{-3}	(7.80± 1.08) x 10^{-3}	(7.94 ± 1.04) x 10^{-3}	-10.5 ± 6.02	-18.10 ± 3.62	10^6
	⊥	(8.16± 3.75) x 10^{-3}	(8.17± 3.76) x 10^{-3}	(8.16 ± 3.36) x 10^{-3}	-10.7 ± 4.28	-20.00 ± 2.33	10^5
800	||	(9.27 ± 3.64) x 10^{-3}	(1.22 ± 0.57) x 10^{-2}	(1.07 ± 0.48) x 10^{-2}	1.04 ± 8.84	-19.3 ± 2.26	10^5
	⊥	(4.76 ± 2.23) x 10^{-3}	(4.73 ± 2.24) x 10^{-3}	(4.75 ± 2.07) x 10^{-3}	-8.17 ± 1.14	-21.1 ± 1.80	10^5
1000	||	(6.10 ± 2.96) x 10^{-3}	(6.03 ± 2.92) x 10^{-3}	(6.06 ± 2.77) x 10^{-3}	-13.00 ± 5.20	-20.5 ± 3.04	10^5
	⊥	(6.83 ± 3.47) x 10^{-3}	(6.82 ± 3.45) x 10^{-3}	(6.82 ± 3.20) x 10^{-3}	-11.5 ± 6.64	-21.9 ± 5.14	10^5

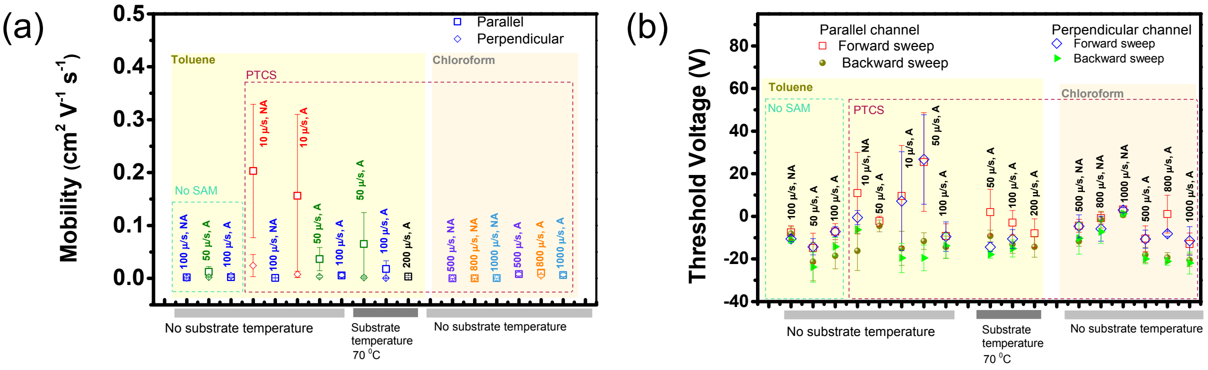

Figure S21. Plots showing (a) the average saturation mobility and (b) average threshold voltage of OFETs fabricated with shear coated, annealed (A) or unannealed (NA) films of DBT-I dissolved in toluene or chloroform, coated at different shearing speeds (10, 50, 100, 200, 500, 800 and 1000 μm/s), with or without using PTCS SAM.
